# Time‐resolved interaction proteomics of the GIGANTEA protein under diurnal cycles in *Arabidopsis*

**DOI:** 10.1002/1873-3468.13311

**Published:** 2018-12-28

**Authors:** Johanna Krahmer, Greg S. Goralogia, Akane Kubota, Argyris Zardilis, Richard S. Johnson, Young Hun Song, Michael J. MacCoss, Thierry Le Bihan, Karen J. Halliday, Takato Imaizumi, Andrew J. Millar

**Affiliations:** ^1^ SynthSys and School of Biological Sciences University of Edinburgh UK; ^2^ Institute of Molecular Plant Sciences University of Edinburgh UK; ^3^ Department of Biology University of Washington Seattle WA USA; ^4^ Graduate School of Biological Sciences Nara Institute of Science and Technology Ikoma, Nara Japan; ^5^ Department of Genome Sciences University of Washington Seattle WA USA; ^6^ Department of Life Sciences Ajou University Suwon Korea

**Keywords:** affinity purification, *Arabidopsis thaliana*, circadian rhythms, flowering time, quantitative mass spectrometry

## Abstract

The plant‐specific protein GIGANTEA (GI) controls many developmental and physiological processes, mediating rhythmic post‐translational regulation. GI physically binds several proteins implicated in the circadian clock, photoperiodic flowering, and abiotic stress responses. To understand GI's multifaceted function, we aimed to comprehensively and quantitatively identify potential interactors of GI in a time‐specific manner, using proteomics on *Arabidopsis* plants expressing epitope‐tagged GI. We detected previously identified (in)direct interactors of GI, as well as proteins implicated in protein folding, or degradation, and a previously uncharacterized transcription factor, *CYCLING DOF FACTOR6* (*CDF6*). We verified CDF6's direct interaction with GI, and ZEITLUPE/FLAVIN‐BINDING, KELCH REPEAT, F‐BOX 1/LIGHT KELCH PROTEIN 2 proteins, and demonstrated its involvement in photoperiodic flowering. Extending interaction proteomics to time series provides a data resource of candidate protein targets for GI's post‐translational control.

## Abbreviations

**3F6H**, 3xFLAG‐6xHis

**MS**, mass spectrometry

**PCA**, principal component analysis

**TAP**, tandem affinity purification

**ZT**, Zeitgeber time

*Arabidopsis thaliana* plants have well‐documented 24‐h rhythms in many physiological processes, from hypocotyl elongation to photosynthetic functions as well as defense responses against pathogen and herbivore attack 1, 2. The overt circadian rhythms are driven by intricate transcriptional‐translational feedback loops 2. Detailed dynamic models based mostly upon transcriptional repression recapitulate the rhythmic expression profiles of these clock genes, including manipulations of the system in mutant plants and under changing photoperiods 3, 4, 5, 6.

Gene expression switches can operate on a timescale of minutes. However, it does not obviously explain the slow 24‐h timescale of circadian clocks. In addition to chromatin modification 7, the slow degradation rate of the transcriptional repressors extends the timescale of transcriptional regulation, which mathematical analysis has long identified and experiments have confirmed 8, 9. In *Arabidopsis*, regulated protein degradation is also crucial to circadian timing and provides one mechanism for environmental light signals to control the pace of the clock 10.

One of the central proteins that regulate the degradation rate of circadian clock proteins in the *Arabidopsis* clock is the GIGANTEA (GI) protein. The *gi* mutants were originally identified as delayed‐flowering mutants under long‐day conditions where wild‐type plants flower early 11, 12. The *gi* mutants also alter the pace of the circadian clock 13, 14, 15, 16, 17. GI affects the clock through interaction with the F‐box proteins of the ZEITLUPE (ZTL)/FLAVIN‐BINDING, KELCH REPEAT, F‐BOX 1 (FKF1)/LIGHT KELCH PROTEIN 2 (LKP2) family, and increases the degradation of the evening‐expressed circadian repressors, TIMING OF CAB EXPRESSION 1 (TOC1), and PSEUDO RESPONSE REGULATOR 5 (PRR5) 18, 19, 20. The degradation is directly mediated by the ZTL/FKF1/LKP2 proteins, together with ARABIDOPSIS SKP1‐LIKE (ASK) and CULLIN (CUL) proteins, to form an SKP‐CUL‐F‐box (SCF) ubiquitin ligase complex that targets these clock proteins to the proteasome 19, 20, 21, 22. ZTL binds to GI in a light‐dependent manner 18. This interaction stabilizes both ZTL and GI. ZTL is thought to not only enhance GI stability but also to sequester GI in the cytoplasm. GI is rhythmically expressed due to circadian control of *GI* transcription, and therefore GI confers rhythmicity upon ZTL protein levels 23, 24, 25. *GI* mRNA levels peak 8–10 h after dawn 14, 26, 27, before repression by the evening complex, which are composed of EARLY FLOWERING 3 (ELF3), ELF4, and LUX ARRHYTHMO (LUX, also termed PHYTOCLOCK1) 26, 28. GI protein interacts with and is destabilized by ELF3 and COP1 29. Within the nucleus, the clock protein ELF4 interacts with GI and sequesters it away from the promoter of the floral induction gene *CONSTANS* (*CO*), contributing to rhythmic regulation of *CO* 24.

The rhythm of *CO* expression provides part of the timing function required to distinguish long days from short days. CO protein activates transcription of *FLOWERING LOCUS T* (*FT*) 30. GI physically associates with the promoter regions of *CO* and *FT* 31, 32, and also binds to transcriptional regulators of *CO* 33. Morning‐expressed *CYCLING DOF FACTOR 1* (*CDF1*)*, CDF2, CDF3,* and *CDF5* directly repress *CO* and *FT* transcription, delaying flowering in long days 34, 35, 36. The F‐box protein FKF1 is co‐expressed with GI, binds both to GI and to CDF1‐CDF5 under light conditions, and initiates the degradation of CDFs by ubiquitination 34, 35. Thus, GI facilitates expression of *CO* and *FT* at the end of long days, by relieving CDF repression 31, 35, 36.

In addition to its roles in the clock and flowering, *GI* has been linked to carbon metabolism, 37, 38, 39 and various stress responses. GI confers tolerance to high salinity through interaction with the protein kinase SALT OVERLY SENSITIVE 2 (SOS2) 40 and is involved in ELF under drought conditions 41. Moreover, mutations in *GI* increase resistance to oxidative stress 42 and freezing 43 due to increased CDF expression levels 44. GI's biochemical mechanisms in most of these responses are unknown.

GIGANTEA's role in the clock is mediated at the biochemical level by co‐chaperone activity, which involves binding to HEAT SHOCK PROTEIN 90 (HSP90) and appears to stabilize ZTL 45, 46. This activity can affect other test substrates but its other native targets, if any, are unknown. As outlined above, GI's known functions with the ZTL family and SOS2 are mediated by protein‐protein interactions. Therefore, GI has been suggested to serve as a scaffold or hub protein that orchestrates other protein interactions 47, for example to provide chaperone activity 46.

Although such protein interactions are thought to mediate GI's functions, these interactions have not been comprehensively and quantitatively analyzed. We therefore conducted interaction proteomics assays using the GI protein, and obtained time‐resolved data on potential direct and indirect partners of GI, over the daily time course. Here, we discuss the abundance profiles of proteins co‐immunoprecipitated with GI, and functions of new candidate interactors and highlight a DOF protein, which we refer to as CDF6, validating its direct interactions and functional importance.

## Materials and methods

### Generation of plant materials

To generate plants with epitope‐tagged GI protein, *gi‐2* mutants were transformed with a construct expressing C‐terminal 3xFLAG‐6His‐tagged GI protein (GI‐3F6H). The full‐length *GI* cDNA without the stop codon was amplified and inserted into pENTR/D‐TOPO vector (Invitrogen, Carlsbad, CA, USA). After sequence verification, the *GI* cDNA was transferred into the pB7HFC vector, designed for in‐frame epitope fusion 48 by a Gateway cloning reaction (Invitrogen). pB7HFC‐GI‐3F6H was introduced to the *gi‐2* mutant by *Agrobacterium*‐mediated transformation. Transgenic plants that rescued the *gi‐2* phenotype were selected, and the expression of the GI‐3F6H protein was verified by western blotting (as in Fig. S1; Methods S1). Samples for the preliminary and qualitative GI tandem affinity purification (TAP)‐mass spectrometry (MS) studies were prepared as described in 49.

To generate *SUC2:HA‐CDF6* plants, the *CDF6* CDS (AT1G26790) was PCR‐amplified using cDNA derived from 2‐week‐old long‐day grown plants as a template, and cloned into pENTR D‐TOPO (Invitrogen), to form pENTR HA‐CDF6. 2.3 kbp of the *SUC2* 5′ upstream promoter region was amplified and cloned into the pENTR 5′‐TOPO vector (Invitrogen), to form pENTR 5′ SUC2. Using a sequential LR clonase II reaction (Invitrogen), we integrated the pENTR 5’ SUC2, pENTR HA‐CDF6 into the R4pGWB501 vector 50, to form *SUC2:HA‐CDF6*. After confirming the sequence, this vector was transformed into Col‐0 WT plants using by *Agrobacterium*‐mediated transformation. Transgenic plants were selected based on the expression level of *CDF6* transcript.

### Plant growth conditions

For flowering time experiments, seeds were sown and stratified at 4 °C for 3 days on soil (Sunshine Mix #4; Sun Gro Horticulture, Agavam, MA, USA), containing Osmocote Classic time‐release fertilizer (Scotts, Marysville, OH, USA) and Systemic Granules: Insect Control (Bionide, Oriskany, NY, USA). Plants were grown at 22 °C under long‐day conditions (16 h light, full‐spectrum white fluorescent light bulbs (F017/950/24” Octron; Osram Sylvania, Wilmington, MA, USA, 70–80 μmol·m^−2^·s^−1^). Flowering time was measured as the mean number of rosette leaves, for at least 16 plants per genotype, ± the standard error of the mean (SEM).

For qPCR analysis, 10‐day‐old seedlings were grown on 1× Linsmaier and Skoog media (Caisson, Smithfield, UT, USA), supplemented with 3% (w/v) sucrose and 0.8% (w/v) agar, under long‐day conditions at 22 °C in growth chambers (CU‐36L5; Percival Scientific, Perry, IA, USA; lighting conditions as for flowering time) and harvested at 3‐h intervals from 1 h after dawn [Zeitgeber time 1 (ZT1)].

For the preliminary TAP‐MS study, growth conditions were the same as for the time series study (see below), and plants were harvested at ZT8. For the qualitative study, plants were grown on soil in long‐day conditions (16 h light, 8 h dark) and harvested at ZT13 on day 14. For the TAP‐MS time series, GI‐3F6H and Col‐0 WT seeds were surface‐sterilized for 10 min with 30% bleach, 0.01% Triton X‐100, followed by four washes with sterile water. After cold‐treatment at 4 °C for 5 days, seeds were grown on agar plates [2.15 g·L^−1^ Murashige & Skoog medium Basal Salt Mixture (Duchefa Biochemie, Haarlem, The Netherlands), pH 5.8] in Percival incubators (CLF Climatics) for 17 days at 85 μmol·m^−2^·s^−1^ (full‐spectrum white fluorescent bulbs) and 21 °C in short‐day conditions (8 h light, 16 h dark). Seedlings were transferred to soil, for 20 days in the same conditions with a light intensity of 110 μmol·m^−2·^s^−1^. Starting at 7 h after dawn, 80 rosettes without roots were harvested for each replicate, in quintuplicates at time points shown in Fig. 2A and flash‐frozen in liquid nitrogen. Dim green safelight was used to harvest samples during darkness. The same total number of WT control samples were harvested as GI‐3F6H replicates at each time point, spread out across the time series (leaving out ZT15 and ZT31).

### Quantitative PCR (qPCR) analysis

Seedlings were ground into powder with a mortar and pestle with liquid nitrogen, and total RNA was isolated by using an illustra RNAspin Mini kit (GE Healthcare, Chicago, IL, USA) according to the manufacturer's instructions. Two microgram of total RNA was reverse‐transcribed using the iScript cDNA synthesis kit (Bio‐Rad, Hercules, CA, USA) according to the manufacturer's instructions. cDNA was diluted five times with water, and 2 μL was used as a template for quantitative PCR (qPCR) analysis using primers as shown in Table S1. *ISOPENTENYL PYROPHOSPHATE/DIMETHYLALLYL PYROPHOSPHATE ISOMERASE 2* (*IPP2*) was used as an internal control for normalization. The average value from WT was set to 1.0 to calculate the relative expression of other lines. To amplify *CO* and *CDF6*, three‐step PCR cycling program was used: 1 min at 95 °C, followed by 40–50 cycles of 10 s at 95 °C, melting temperatures for 15 or 20 s, and 72 °C extension for 15 s. To amplify *GI*,* FT*, and *IPP2*, a two‐step PCR cycling program was used: 1 min at 95 °C, followed by 40–50 cycles of 10 s at 95 °C and 20 s at 60 °C. Data show the average of three biological replicates with SEM; each measurement had two technical replicates.

### Protein Extraction and tandem affinity purification (TAP) procedure

All steps in the protein extraction, TAP, and preparation for MS were carried out in random sample order to avoid bias due to order of processing. Frozen plant tissue was ground to a fine powder in a liquid nitrogen and dry ice‐cooled mortar and processed essentially as described 49. Detailed procedures are described in Supporting Experimental Procedures (Methods S2).

### Protein digestion and mass spectrometric analysis

Preparation of samples for MS for the qualitative and time series studies analysis used an on‐bead digest, prior to mass spectrometric analysis. Detailed procedures are described in Supporting Experimental Procedures (Methods S3).

### Proteomics data analysis and bioinformatics

For the qualitative study, database searches were performed using Comet 51, searching against the Uniprot *Arabidopsis* protein sequence database, and using Percolator (Matrix Science, Boston, MA, USA) with a *q*‐value cutoff of 0.01. Cysteine residue masses were considered statically modified by iodoacetamide, and methionine dynamically modified by a single oxidation. Precursor mass tolerance was 10 p.p.m., and product ion tolerance was 0.5 Da. The principle of parsimony was used for protein inference, and at least two unique peptides were required for each identified protein.

The time series data were analyzed using the commercial Progenesis LC‐MS software (version 4.1.4924.40586; Nonlinear Dynamics, Newcastle, UK) for label‐free quantitation. Raw files were imported into a label‐free analysis experiment, chromatograms were subjected to automatic alignment and peak picking. Only charges 2+, 3+, and 4+ and data from 25 to 75 min of the runs were chosen for analysis. The exported file of MS/MS spectra was uploaded on the Mascot website (version 2.4) and a search was carried out with the following parameters: database Arabidopsis_1rep (version 20110103), trypsin as enzyme, allowing up to two missed cleavages, carbamidomethyl (C) as a fixed modification, Oxidation (M), Phospho (ST) and Phospho (Y), as variable modifications, a peptide tolerance of 10 p.p.m., and MS/MS tolerance of 0.05 Da, peptide charges 2+, 3+, and 4+, on a QExactive instrument (Thermo, Waltham, MA, USA), and with decoy search to determine false discovery rate (FDR). For export, an ion‐cutoff of 20 was chosen (exported peptide measurements: Data S9). The MS proteomics data have been deposited to the ProteomeXchange Consortium (http://proteomecentral.proteomexchange.org) *via* the PRIDE partner repository 52 with the dataset identifier PXD006859. Technical outliers were identified using correlation analysis and principal component analysis (PCA) of protein abundance data implemented by an R script (Data S5). The average Pearson correlation coefficient of each GI‐TAP replicate with the other replicates of the same time point was above 95% for all GI‐3F6H samples apart from sample 19E (Fig. S2), which was also clearly separated from all other samples by PCA and was therefore discarded.

A custom R script performed further statistical analysis and plotting (Data S7). We used a *t*‐test to determine for each protein, whether the maximum GI‐TAP time point (omitting the 31‐h time point) is significantly different from the WT control average using *q*‐values [Benjamini‐Hochberg (BH) corrected *P*‐values]. ‘Fold enrichment’ is the ratio of the highest GI‐TAP time point to the WT control average gives. To assess temporal changes, ANOVA was performed on arcsinh‐transformed GI‐TAP data, including the ZT 31 time point. To assess rhythmicity, we used the JTK_CYCLE tool 53 to analyze periods of 22–26 h (Data S8). The summary heatmap (Fig. 2D) used the heatmap.2 function of the pvclust v2.0 R package 54. Gene ontology (GO) analysis was performed using topgo (http://bioconductor.org/biocLite.R, version: 2.16.0, 55, 56, using a node size of 3, as described by 57 (Data S6). Biological context was provided by subcellular locations annotated in the SUBA resource 58 and interaction data in the BioGrid 59.

### Yeast two‐hybrid assay

Full‐length *CDF6* coding sequence was PCR‐amplified using cDNA as template with primers shown in Table S1, cloned into pENTR/D‐TOPO (Invitrogen) and sequence‐verified. The plasmid cassette was transferred to pAS‐GW, a gateway compatible bait vector 60 using LR clonase II (Invitrogen). The GI‐FL, GI‐N, GI‐M + C, FKF1, LKP2, and ZTL clones used in this analysis were described previously; GI‐FL, GI‐N and GI‐C 31, and FKF1, LKP2, and ZTL 34. Yeast strains Y187 and AH109 were transformed with prey and bait vectors, respectively using the standard yeast transformation protocol (Clontech, Mountain View, CA, USA). After colonies formed on –W or –L containing media, three independent colonies were grown together, and then mated against their corresponding pairs for 3 days on YPDA media. After mating, yeast colonies were transferred onto –WL media. After checking for mating confirmation, yeast sectors were retransferred at the same time onto –WL and –WLH media. The experiments were repeated several times with the same results.

### Modeling methods

**Figure 1:**
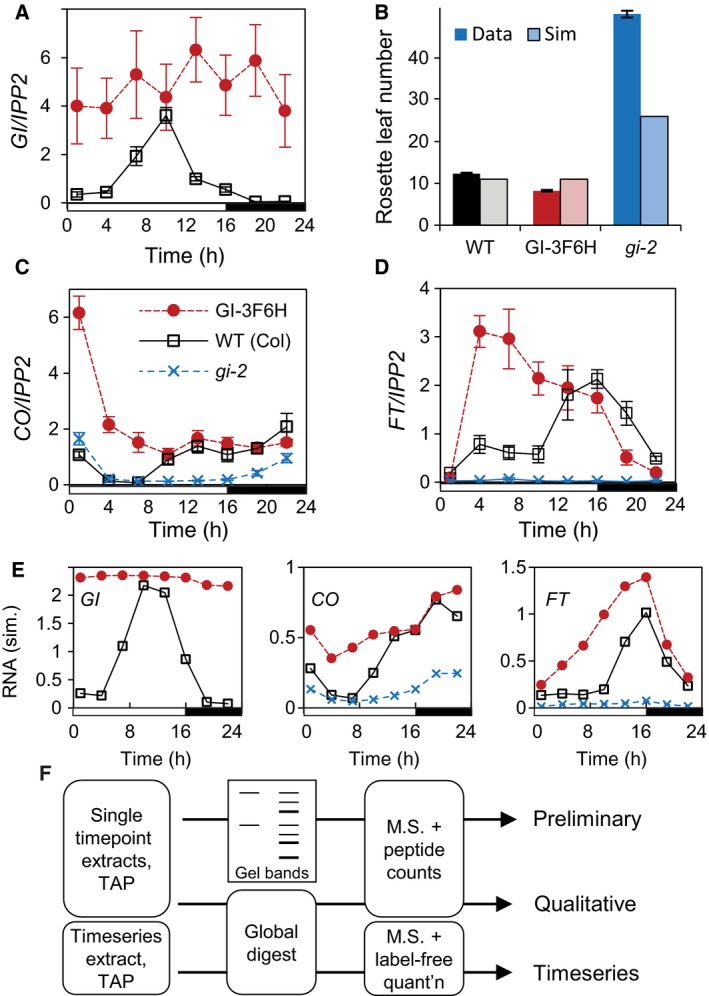
Evaluation of the *GI‐3F6H* complementation line. (A,C,D) mRNA expression was tested in samples of *Arabidopsis* plants of the Col‐0 WT (black open squares), *gi‐2* mutant (blue crosses) and *gi‐2* plants constitutively expressing the GI‐3F6H fusion protein (red filled circles) under long‐day conditions. qPCR assays detected *GI* (A), *CO* (C) or *FT* (D) mRNA. Data are means of biological triplicates, normalized to an *IPP2* internal control; error bar, SEM. (B) Rosette leaf number was measured (data in bold) at flowering time in GI‐3F6H, with WT and parental *gi‐2* mutant controls, under long‐day conditions. The flowering experiment was simulated using the FMv2 (pale colors). Data are averages of 16 plants; error bar, SEM. (E) *GI* transcription in the FMv2 was adjusted to match the *GI* mRNA profile of GI‐3F6H in (A); predicted expression profiles of *CO* and *FT* are shown (as in C, D). (F) Overview of proteomics studies using GI‐3F6H.

Simulations of the P2011 clock model 5 for *GI* and GI‐3F6H were performed in COPASI v4.16 61. Simulations of the Framework Model FMv2 62 for *CO*,* FT,* and flowering time were performed in MATLAB (Mathworks, Cambridge, UK). Both models are available online: P2011 (http://www.plasmo.ed.ac.uk/plasmo/models/download.shtml?accession=PLM_71&version=1) and FMv2 (https://fairdomhub.org/models/248?version=2). The higher arrhythmic *GI* RNA levels in *GI‐3F6H* plants were simulated by reducing the affinity of *GI* for its rhythmic transcriptional inhibitors (parameters *g14, g15*) by 100‐fold each, compared to the default, wild‐type values. Transcriptional activation (parameter *n12*) was then reduced by 36% to match the observed *GI‐3F6H* mRNA level. The effects on other model readouts (Figs 1 and 3) were caused by this simulated transcriptional mis‐regulation of *GI*. Simulations of the flowering pathway were conducted using the photoperiod and temperature conditions of the corresponding experiments.

## Results

### Characterization of the GI‐3F6H transgenic plant line

We transformed the strong *gi‐2* mutant (a deletion allele predicted to truncate ~ 90% of GI protein) 14 with a construct to express 3xFlag‐ and 6xHis‐tagged GI protein under the control of the CaMV *35S* promoter (GI‐3F6H). We aimed to express GI‐3F6H protein constitutively at a similar level throughout the day to be able to immunoprecipitate a similar amount of GI at each time point 31. This will enable us to detect the changes in interaction of certain proteins with GI rather than the changes in the amount of co‐immunoprecipitated proteins caused by the different amount of GI expressed. After isolating several positive transformants, we chose the line in which the expression levels of *GI‐3F6H* transcripts were similar to the peak expression levels of the endogenous *GI* (Fig. 1A). As a first experiment, we performed a preliminary study where we used TAP of GI‐3F6H followed by silver‐staining of a protein gel separation and LC‐MS of excised gel bands (Fig. 1F, Fig. S1, Data S1). Our GI‐3F6H line expressed sufficient GI protein for effective TAP and analysis of gel bands by MS identified GI and known interactors (Fig. S1, Data S1). In addition, this GI‐3F6H construct completely rescued the late flowering phenotype of *gi‐2*, indicating that GI‐3F6H is functional (Fig. 1B). In the GI‐3F6H line, *CO* and *FT* mRNAs were higher than in the WT in the morning (Fig. 1C,D), consistent with activation of these flowering‐promoting genes by GI in the light 31, 32. This was also reflected by slightly ELF of the GI‐3F6H plants relative to the WT (Fig. 1B).

GIGANTEA has multiple, known effects on the clock and flowering genes and proteins; several of these effects have been incorporated into mathematical models 5, 62. In order to test whether the effects of the mis‐regulated *GI‐3F6H* transgene were replicated by these known mechanisms, we simulated the rescued mutant line in the *Arabidopsis* Framework Model version 2 62, a mechanistic, mathematical model that includes photoperiodic flowering (Fig. 1B,E). The WT and *gi‐2* simulations closely matched the mRNA data. This data set favored morning (1–4 h after dawn) expression of *CO* and *FT* compared to evening (13–16 h) expression slightly more than the model, possibly reflecting a reduced *GI* mRNA level at 13 h in this data set compared to previous training data. The model correctly predicted an elevated *CO* mRNA level at 1 h in GI‐3F6H plants, though the observed level was ~ 5‐fold rather than ~ 2‐fold higher (Fig. 1C). This *CO* peak induced more *FT* at 4 h in the model (Fig. 1E) than in the plant (Fig. 1C), and therefore earlier flowering (Fig. 1D). Morning regulation of *FT*, in the presence of unusually high GI levels, differed most between the model and the plant, highlighting this as an area for future model refinement.

**Figure 2:**
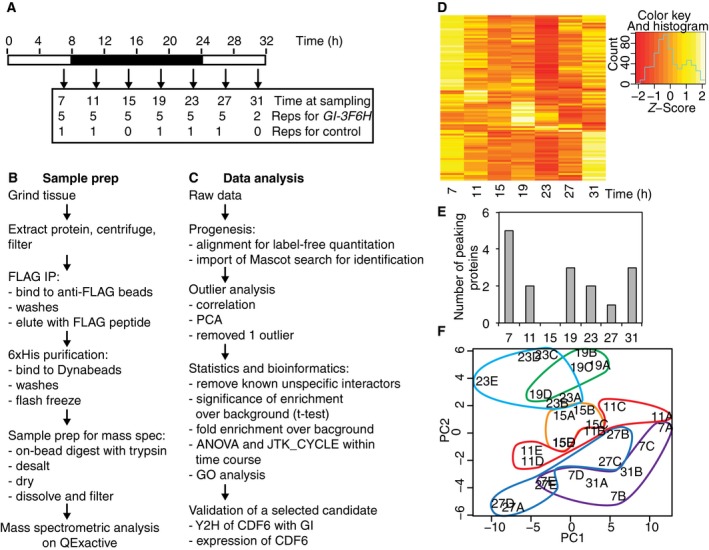
GI‐TAP time series. (A) Samples from GI‐3F6H transgenic plants and WT control plants grown in short‐day conditions were harvested at the indicated time points and replication. (B) Workflow for protein extraction, TAP using FLAG and His tags, and peptide preparation for MS. (C) Workflow for label‐free, quantitative data analysis, statistical and bioinformatics tests. (D) Heat map of protein abundance over time for 88 proteins with significant enrichment of at least two‐fold (excluding proteins that bind to GFP or are only found in inaccessible compartments). (E) Distribution of peak times, for 16 proteins shown in (D) with significant change over the GI‐3F6H time course (ANOVA *q* < 0.05 or JTK_CYCLE *q* < 0.05). (F) PCA separates GI‐TAP time points (grouped by number and contour, with replicate letter, all quantified proteins used) over components 1 and 2 (PC1, PC2). Note 31 h time point replicates 7 h.

Next, GI‐3F6H and WT plants (control) were grown in long days for 2 weeks and harvested at ZT 13 when GI protein peaks 31 and potential GI‐interacting proteins were identified in a qualitative proteomics study (Fig. 1F). TAP of GI‐3F6H was carried out, followed by on‐bead digestion and qualitative MS analysis (Fig. 2B), reporting peptide counts (Fig. 1F). Fifty *Arabidopsis* proteins were identified by at least one peptide in each of the GI‐3F6H samples and none in the controls. In order to exclude known nonspecific interactors, we eliminated all proteins from our list that were previously reported to be purified by GFP‐3F6H with the same sample preparation protocol 48 (Data S3). In addition, during the extraction, GI‐3F6H can come in contact with proteins from compartments that are inaccessible to it in an intact cell. Therefore, we also excluded proteins from further analyses that are located in the chloroplast or mitochondria but not in the cytoplasm or nucleus according to their GO annotation 63 (Table 1; full results in Data S2). Eighteen proteins remained after this background removal strategy, indicating the potential to identify previously undiscovered interactions, as discussed below. Detection of known direct interactors, such as ZTL, FKF1, and LKP2, as well as indirect interactors, ASK1 and ASK2 (direct interactors of ZTL and FKF1) validated the methodology 16, 21, 31, 64.

**Table 1:** Candidate interacting proteins identified in the qualitative study. Control (WT samples) and GI‐3F6H samples were extracted in RIPA or SII buffer. Eighteen *Arabidopsis* proteins were identified by at least one peptide in each GI‐TAP sample and none in the WT background controls, excluding proteins that are likely contaminants as they bind to GFP‐3F6H 48 or localized to other compartments than GI (chloroplast, mitochondria). The right–hand columns cross‐reference the time series study (Table 3), with fold enrichment and significance (*q*‐value) of the maximum GI‐3F6H time point relative to the WT control. Bold: known direct or indirect interactors and homologs. n.d.: not detected

Accession	Name	Number of peptides identified in qualitative TAP experiment		GI‐3F6H time series enrichment (max. GI/WT)	
		GI TAP RIPA	GI TAP SII	*t*‐test *q*‐value	Fold enrichment
**AT1G22770**	**GIGANTEA**	**309**	**299**	**0.00030**	**76**
**AT5G57360**	**ZTL**	**47**	**61**	**0.00020**	**32**
AT5G06600	UBP12	15	20	0.00022	19
AT3G13920	EIF4A1	5	10	n.d.	n.d.
**AT1G68050**	**FKF1**	**6**	**9**	**0.00038**	**12**
**AT2G18915**	**LKP2**	**6**	**9**	**0.00074**	**13**
**AT1G75950**	**ASK1**	**7**	**8**	**0.0011**	**8.6**
AT3G08530	CHC2	1	5	0.43^a^	2.44
AT5G60390	EFTu/EF1‐A	4	2	n.d.	n.d.
AT2G44060	At2G44060	3	2	n.d.	n.d.
**AT5G42190**	**ASK2**	**2**	**2**	n.d.	n.d.
AT3G56340	RPS26E	1	3	n.d.	n.d.
AT4G03550	CALS12	1	1	n.d.	n.d.
AT1G70490	ARF2‐B	1	1	n.d.	n.d.
AT1G80870	AT1G80870	1	1	n.d.	n.d.
AT2G29420	GSTU7	1	1	n.d.	n.d.
AT3G58350	RTM3	1	1	n.d.	n.d.
AT5G23540	AT5G23540	1	1	0.17^a^	1.88^a^

a Below threshold in time series study.

### Candidate GI‐interacting proteins from time series data

Although GI plays an important role in photoperiodic flowering in long days, the function of GI under short‐day conditions remains elusive. Therefore, we grew plants in short days to identify uncharacterized interactors of GI potentially involved in other responses. In order to obtain time‐resolved interaction data, we applied the same GI‐TAP method as in the qualitative study to plants grown in short‐day conditions, at six time points in biological quintuplicate, with additional duplicate samples at time point 31 h (replicating the 7‐h time point; Fig. 1F ‘time series’, Fig. 2A). Short‐day conditions ensure plants to be at a vegetative stage at the time of sampling, while being large enough to obtain sufficient amounts of tissue from a manageable number of plants for our time‐resolved TAP‐MS procedures. Extraction, TAP, and sample preparation for MS were carried out as for the qualitative analysis (Fig. 2B). Using the Mascot search engine to identify peptides, our choice of peptide score cutoff of 20 resulted in an FDR of 0.023. After identification and quantification of proteins (Fig. 2C), one outlier (GI‐3F6H sample at ZT19, replicate E) was excluded from subsequent analysis (see Experimental Procedures; Fig. S2). PCA of the remaining GI samples maximally separated the mid/late‐night time points 19 h and 23 h from mid‐day time points 7 h and its replicate 31 h (Fig. 2E).

Two thousand three hundred thirty‐six peptides were detected in the time series study, from which 500 proteins were quantified (Table 2). In order to exclude known unspecific interactors, we used the same strategy as for the qualitative study, eliminating 80 proteins previously purified by GFP‐3F6H 48 and 169 chloroplast and mitochondrial proteins 63 (Data S3, S4 and S6). The analytical methods also quantified the identified peptide peaks in WT control samples (one replicate for each time point except ZT 15 and ZT 31) that had been subjected to the same TAP procedure (Figs 1F and 2A). Subsequent analysis used raw abundance data exported from Progenesis as opposed to the abundance which is normalized by the sum of all intensities of ions with the chosen inclusion criteria (see Materials and methods) in each mass spectrometric analysis; however, analysis of normalized data gave very similar results (data not shown and Data S3). The fold enrichment of each protein in the GI‐3F6H was calculated as the peak GI‐3F6H abundance relative to the average abundance in the WT control samples. Potential interacting proteins were identified as significantly enriched by *t*‐test compared to the WT control, with a significance threshold adjusted for multiple testing (BH‐adjusted *q*‐value < 0.05). Fold‐enrichment threshold values were informed by the results for known interactors. The direct interactors ZTL, FKF1, and LKP2 were more than 10‐fold enriched over the WT control in the GI‐3F6H time series. Indirect interactors CUL1/CUL2 and GLUTAMINE SYNTHETASE 2 (GLN2) 21, 49 were two‐ to three‐fold more abundant at their peaks than the time series control and were not identified in the preliminary or qualitative studies. Hereafter, we refer to significantly enriched proteins with at least four‐fold enrichment as highly enriched (55 proteins) and to proteins with two‐ to four‐fold enrichment as weakly enriched (a further 88 proteins).

**Table 2:** Numbers of quantified and significant proteins in the GI‐3F6H‐MS time series. The raw data of the Progenesis output file and the raw data after removing likely unspecific proteins (GFP interacting proteins 48) and plastid or mitochondrial proteins, (see Data S3) was used for statistics

	1 or more unique peptides	Of these, JTK_CYCLE *q*‐value < 0.05	2 or more unique peptides	Of these, JTK_CYCLE *q*‐value < 0.05
Total quantifiable identifications	500	43	231	17
Without GPF interactors	420			
Without plastid/mitochondrial proteins	251	32	88	9
Of these 251 (1 or more unique peptides) or 88 (2 or more unique peptides):				
Significantly enriched (BH *q*‐value < 0.05)^a^	91	10	36	3
And fold enrichment > 2	88	10	33	3
And fold enrichment > 4	55	8	18	2

a These numbers exclude proteins where t‐test was impossible due to missing quantifications.

**Figure 3:**
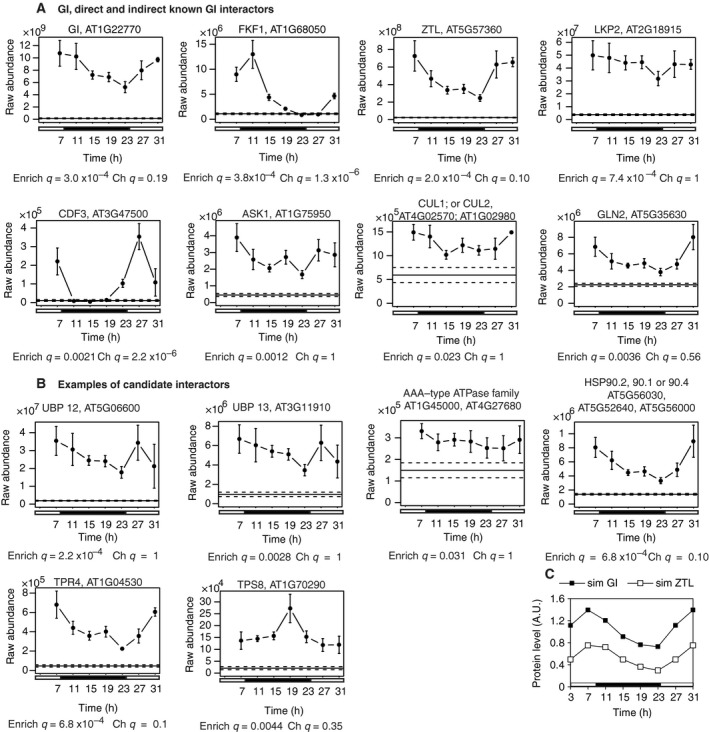
Diel profiles of immunoprecipitated GI and interacting proteins. Immunoprecipitated protein abundance of (A) GI and direct and indirect interactors detected in the time series study, along with (B) HSP90 and candidate direct or indirect interactors. Multiple gene identifiers indicate related proteins were not distinguished by the three CUL or five HSP90 peptides detected. GI‐3F6H samples, markers; error bar, SEM. Average of WT control, horizontal line, ± SEM, dashed line. Time (*T*); Significance of enrichment and temporal change are shown, as *q*‐values of *t*‐test comparing GI‐TAP peak to WT (Enrich *q*) and of JTK_CYCLE within the GI TAP‐MS time series (Ch *q*). Biological replicates in (A and B):: 5, except for ZT 19 (four replicates) and ZT 31 (two replicates). (C) Simulation of the GI and ZTL protein time series, using the model matched to *GI:3F6H* RNA data (Fig. 1E) under short‐day conditions, closely matches observations (panel A).

Among these 88 proteins, 13 changed significantly in abundance within the time series (assessed for any change by ANOVA, *q*‐value < 0.05) or 10 as assessed for rhythmic profiles (by JTK_CYCLE *q*‐value < 0.05; Table 2 and Data S3; Data S8). Peak abundance for most of the 16 proteins with changing or rhythmic precipitated protein abundance, as well as nonrhythmic/changing precipitated proteins occurred at 7 h, with lowest average abundance at 23 h among the significantly enriched proteins, and no rhythmic or changing proteins peaking at 15 h (Fig. 2D,E). The immunoprecipitated abundance of GI changed about two‐fold over time (Fig. 3A). The observed abundance changes for immunoprecipitated GI and ZTL closely matched the predicted protein profiles (Fig. 3C) from simulation of GI‐3F6H (as in Fig. 1E). This result indicated that the multiple mechanisms of GI and ZTL protein regulation in the model were sufficient to replicate the abnormal accumulation of GI‐3F6H protein, and its effects on ZTL. The change in precipitated GI abundance was not significant by ANOVA or JTK_CYCLE.

### Functional categorization of GI‐TAP enriched proteins

Gene ontology analysis was done, using the candidates in Table 3 as foreground. GO terms related to protein degradation were overrepresented among the candidates, as well as light response related terms, some metabolic processes, and flower development (Data S4 and S6).

**Table 3:** Known and candidate interactors of GI from GI‐3F6H TAP‐MS time course experiment. Quantified proteins with two or more peptides that were significantly enriched (in t‐test of maximum GI‐TAP time point with WT control *q*‐value < 0.05) by at least two‐fold (max. GI‐TAP/WT > 2), ranked by fold enrichment. Only FKF1, CDF3 and CDF6 were rhythmic (JTK_CYCLE *q*‐value < 0.05). Where peptides matched very similar proteins, multiple accession numbers are shown. Bold type, known direct or indirect interactors. Detection in the preliminary study (Prelim., Fig. 1F) is shown, and the sum of peptide numbers detected in the qualitative study (Qual., Fig. 1F) in GI‐3F6H and WT (control). Selected proteins detected by single peptides are shown below, along with proteins suggested by other hypotheses (see Discussion) that were below thresholds in the time series (*) but were detected in the qualitative study. Likely unspecific interactors 48 and inaccessible proteins are left out in this table

Accession	Name	Quantitative enrichment (max GI TAP/control)					
		Time series, number of peptides	*t*‐test (*P*‐value)	*t*‐test BH adjusted (*q*‐value)	Max GI/control	Prelim. studyDetected? (Y/n)	Qual. studyTotal peptides, GI‐TAP/control
≥ 2 peptides per protein							
**AT1G22770**	**GIGANTEA**	**59**	**6.2E‐06**	**3.0E‐04**	**76**	**Y**	**608**/**0**
**AT5G57360**	**ZTL**	**29**	**2.5E‐06**	**2.0E‐04**	**32**	**Y**	**102**/**0**
AT5G06600	UBP12	9	3.60E‐06	2.17E‐04	19	Y	35/0
AT1G04530	TPR4	2	2.70E‐05	6.80E‐04	15	Y	n.d.
**AT2G18915**	**LKP2**	**18**	**3.4E‐05**	**7.4E‐04**	**13**	**Y**	**15/0**
AT4G36250	ALDH3F1	2	4.13E‐05	8.30E‐04	12	n	n.d.
**AT1G68050**	**FKF1**	**9**	**1.3E‐05**	**3.8E‐04**	**12**	**Y**	**15/0**
AT5G54770	THI1	2	0.00051	0.0044	11	n	n.d.
AT5G22800	EMB86	3	6.33E‐05	0.0011	11	n	n.d.
AT1G74730	DUF1118	2	0.00031	0.0030	7.6	n	n.d.
AT3G11910	UBP13	7	2.76E‐04	0.0028	6.9	Y	n.d.
AT5G38660	APE1	3	5.27E‐04	0.0044	6.5	n	2/0
AT3G60750; AT2G45290	Transketolase	9	0.0060	0.023	6.5	n	n.d.
**AT5G56030** **;** **AT5G52640** **;** **AT5G56000**	**HSP90.2; HSP90.1; HSP90.4**	**5**	**2.8E‐05**	**6.8E‐04**	**6.4**	**n**	**n.d.**
AT3G02530	TCP‐1/cpn60 chaperonin	2	1.08E‐04	0.0015	5.6	n	n.d.
AT3G03780	MS2	2	5.44E‐04	0.0044	5.6	n	n.d.
AT1G18080	RACK1A	2	4.06E‐04	0.0038	5.1	n	n.d.
AT3G03960	TCP‐1/cpn60 chaperonin	2	0.0022	0.011	5.1	n	n.d.
AT1G07410; AT1G01200	RAB‐A2B	2	0.010	0.032	3.8	n	n.d.
AT1G54270; AT3G19760	EIF4A‐2	2	0.0027	0.013	3.7	Y	n.d.
AT5G46290	KASI	3	0.0014	0.0082	3.6	Y	1/0
AT5G35630	GS2	5	7.13E‐04	0.0054	3.6	n	2/0
AT5G60790; AT3G54540	GCN1	3	0.0088	0.031	3.4	n	n.d.
AT2G28000	CPN60A	2	0.0022	0.011	2.9	n	n.d.
AT1G78570	RHM1	2	0.0014	0.0082	2.8	n	n.d.
AT2G36880; AT1G02500	MAT3	2	0.0013	0.0081	2.8	n	n.d.
AT2G21330; AT2G01140	FBA1	2	0.0022	0.011	2.7	n	n.d.
ATCG00820	RPS19	2	0.0021	0.011	2.6	n	n.d.
AT1G20620	CAT3	11	0.0040	0.018	2.5	Y	n.d.
**AT4G02570** **;** **AT1G02980**	**CUL1**	**3**	**0.010**	**0.031**	**2.5**	**n**	**n.d.**
AT3G42050	Vacuolar ATP synthase subunit H	2	0.0014	0.0082	2.4	n	3/18
AT2G37270	RPS5B	2	0.013	0.037	2.3	n	n.d.
AT2G09990; AT3G04230	Ribosomal protein S5	4	0.018	0.048	2.1	n	n.d.
≥ 1 peptides per protein (selection)							
AT5G10450	GRF6	1	1.3E‐05	3.8E‐04	90	n	n.d.
AT1G60780	HAP13	1	0.011	0.034	58	n	n.d.
**AT3G47500**	**CDF3**	**1**	**2.4E‐04**	**0.0026**	**33**	**n**	**n.d.**
AT1G14510	AL7	1	4.7E‐05	8.7E‐04	18	n	n.d.
AT3G14420.1; AT4G18360	Aldolase‐type TIM barrel	1	0.0089	0.031	17	n	n.d.
AT1G26790	CDF6	1	0.0010	0.0068	15	n	n.d.
AT3G13470	TCP‐1/cpn60 chaperonin	1	0.018	0.048	15	n	4/0
AT1G70290	TPS8	1	5.4E‐04	0.0044	13	n	n.d.
AT5G12140	CYS1	1	2.6E‐04	0.0027	10	n	1/0
AT1G20330	SMT2	1	1.5E‐04	0.0017	9.1	n	n.d.
AT4G11150	TUF	1	0.0013	0.0081	8.8	n	5/10
**AT1G75950**	**ASK1**	**1**	**7.2E‐05**	**0.0011**	**8.6**	**n**	**13/0**
AT2G16570	ASE1	1	0.0082	0.030	8.1	n	n.d.
AT2G33040	ATP3	1	0.0094	0.031	7.8	n	n.d.
AT1G24510	TCP‐1/cpn60 chaperonin	1	7.9E‐04	0.0057	7.3	n	n.d.
AT3G18190	TCP‐1/cpn60 chaperonin	1	1.4E‐04	1.7E‐03	7.0	n	n.d.
AT1G72730	DEA(D/H)‐box RNA helicase	1	6.7E‐05	0.0011	7.0	n	n.d.
AT1G53750	RPT1A	1	0.0096	0.031	6.6	n	n.d.
AT4G31420	REIL	1	0.0020	0.011	6.6	n	n.d.
AT5G28050	Cytidine/deoxycytidylate deaminase	1	1.3E‐04	0.0017	5.1	n	n.d.
AT1G29880	glycyl‐tRNA synthetase	1	0.0010	0.0068	4.9	n	n.d.
AT5G51110	Transcriptional coactivator	1	6.8E‐04	0.0053	4.8	n	n.d.
AT5G01410	PDX1	1	0.0096	0.031	4.7	n	n.d.
AT3G60300	RWD domain‐ containing	1	0.012	0.037	4.4	n	n.d.
AT5G58140	PHOT2	1	0.0087	0.031	3.5	n	n.d.
AT4G38630	RPN10	1	0.014	0.041	3.3	n	n.d.
AT4G25630	FIB2	1	0.0052	0.022	3.0	n	n.d.
AT1G48630	RACK1B_AT	1	0.0059	0.023	2.9	n	n.d.
AT1G56110	NOP56	1	0.014	0.041	2.7	n	n.d.
AT1G20200	EMB2719	1	0.0050	0.022	2.4	n	2/1
AT5G41210	GSTT1	1	0.018	0.048	2.3	n	n.d.
AT3G20050	TCP‐1	1	0.0098	0.031	2.2	n	2/0
AT1G63660	GMP synthase	1	0.0097	0.031	2.2	n	n.d.
AT1G45000.1; AT4G27680	AAA‐type ATPase	1	0.0097	0.031	2.2	n	n.d.
GFP‐TAP binding but found in time series and qualitative study and not background of qualitative study							
AT3G17390	SAM4	2	0.0058	0.016	1.9*	n	3/0
AT5G17920	MS1	4	0.056*	0.095*	1.8*	n	7/0
AT5G02500; AT1G16030; AT1G56410; AT3G09440; AT3G12580; AT5G02490; AT5G28540	Hsp70 family		0.053*	0.26	2.52	n	13/0

### Rhythmic profiles of known interactors

In contrast to the weakly rhythmic trend in abundance of the immunoprecipitated GI protein, known interactors showed contrasting profiles (Fig. 3A). The direct interactors FKF1, ZTL, and LKP2 showed temporal profiles consistent with their mRNA expression patterns 65, 66, 67, 68 (Fig. S3). The ZTL profile paralleled GI, consistent with their mutual stabilization 23 and closely matched by the prediction from the model simulation (Fig. 3C). Co‐immunoprecipitated LKP2 abundance had a similar trend, consistent with arrhythmic mRNA expression of *ZTL* and *LKP2*. Only FKF1 and CDF3 were strongly rhythmic, with FKF1 peaking at 7–11 h, resembling previous data 35, 69 and CDF3 peaking at 27 h. Therefore, CDF3 level is in antiphase with FKF1, in line with the degradation of CDFs by FKF1 34, 35. These results demonstrate the consistency of our data with published results. CDF3 was quantified using a single, individually inspected peptide, indicating that such data should not be excluded from analysis.

Several established indirect interactors of GI were quantified (Fig. 3A). CDF3 and GLN2 are client proteins of FKF1 49, with CDF3 also being a direct GI interactor (see above) 31, whereas ZTL and LKP2 also interact with the core components of the SCF ubiquitin E3 ligase detected here, ASK1 and CUL1 and/or CUL2 (closely related proteins that were not distinguished by the peptides detected).

### The predicted functions of candidate interactors include protein degradation and stabilization

In addition to verifying the known indirect interactor CUL1, our analysis enriched other proteins involved in protein stability (Fig. 3B). The ubiquitin‐specific proteases (UBP) 12 and UBP13 (AT5G06600 and AT3G11910) were enriched in the time series and qualitative studies. Both UBP12 and UBP13 regulate the period length of the circadian clock as well as photoperiodic flowering 70, therefore the function of UBP12 and UBP13 in the clock and flowering regulation might be through the GI complex (see note added in proof). AAA‐type ATPase family proteins related to components of the 26S proteasome (AT1G45000 and/or AT4G27680) and a protease inhibitor, CYSTATIN 1 (AT5G12140) were also enriched. Several proteins annotated as being involved in protein stabilization were also significantly enriched by GI‐3F6H, such as Cpn60 chaperonin family proteins (AT1G24510, AT3G18190, AT3G02530 and AT3G03960), the putative co‐chaperone TPR4 (AT1G04530; Table 3, Fig. 3B) and at least one HSP90 (AT5G56030, AT5G52640 and/or AT5G56000, Fig. 3B). HSP70 family proteins were just below the enrichment cutoff (Table 3).

In contrast, neither additional F‐box proteins nor other proteins involved in circadian timekeeping were identified as strong candidate interactors (enrichment of PRR3 in the time series was below the significance threshold). Multiple, metabolic enzymes and translation elongation or initiation factors were enriched in the GI‐3F6H time series. Among those (Table 3), were GTP‐binding translation factors (AT1G72730 and AT1G54270 and/or AT3G19760), TREHALOSE‐6‐PHOSPHATASE SYNTHASE 8 (TPS8; Fig. 3B), a phosphofructokinase family protein (AT1G20950) and a GMP synthase homolog (AT1G63660, Table 3) and a pyruvate kinase family protein (AT2G36580). In addition, a protein that binds to di‐or trimethylated histone H3, ALFIN‐LIKE 7 (Table 3) was enriched by GI‐3F6H. The candidate interactors suggest new clients and mechanisms of GI action related to those in other species (see Discussion), though their physiological significance awaits confirmation.

### CDF6 is a GI interactor that contributes to photoperiodic flowering

**Figure 4:**
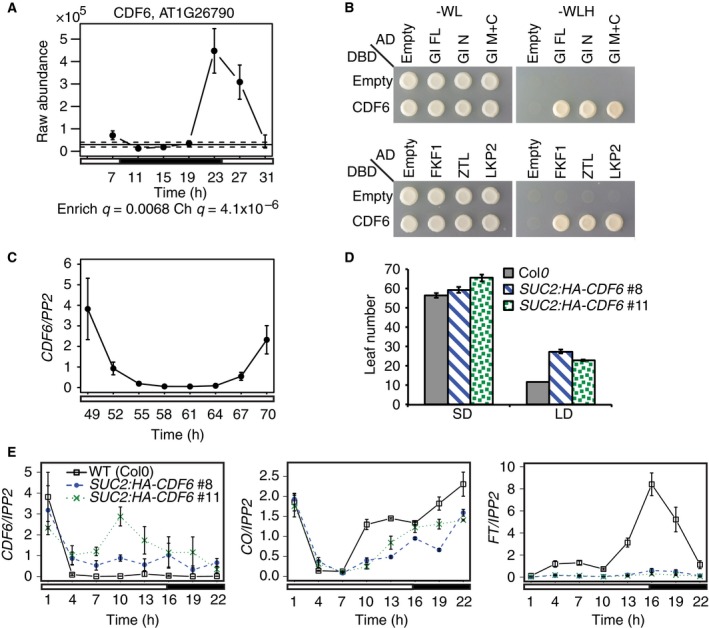
CDF6 interacts with GI and functions in photoperiodic flowering. (A) GI‐interaction profile of CDF6 in the time series study is similar to CDF3 (Fig. 3A). *n* = 5 (except ZT19: *n* = 4) (B) Yeast two‐hybrid assays validate interaction of CDF6 with full‐length GI, N‐ and C‐terminal domains of GI, as well as ZTL, FKF1 and LKP2. AD, activation domain; DBD, DNA binding domain. (C) Circadian expression profile of *CDF6* mRNA, in WT plants 3 days after transfer to constant light, *n* = 3. (D) *CDF6* over‐expression delays flowering of transgenic *SUC2:HA‐CDF6* lines more under long days, compared to WT control, than under short days. Each transgenic line differed significantly from WT,* t*‐test *P* < 0.0001, except #8 in SD, not significant. *n* ≥ 16 (E) mRNA expression profiles of *CDF6, CO* and *FT* were tested by qPCR in WT and the overexpressor lines, confirming that CDF6 suppresses evening *CO* and *FT* expression. *n* = 3.

AT1G26790 encodes a predicted DOF transcription factor that was up to 15‐fold enriched around dawn in our GI‐3F6H time series results (23 h and 27 h; Fig. 4A). This protein was the most significantly rhythmic of the candidate interactors after FKF1 and CDF3 (BH‐adjusted *P*‐value from JTK_CYCLE = 8 × 10^−6^). Its immunoprecipitated protein levels were the most anticorrelated with FKF1 levels among the highly enriched proteins (Fig. 4A), followed by CDF3 (*r* = −0.65 and −0.40, respectively). The DOF protein AT1G26790 is a close homolog of CDF5 35, 71 and its mRNA expression showed a robust circadian oscillation in constant light (Fig. 4C). Therefore, we named this gene *CYCLING DOF FACTOR 6* (*CDF6*). We then validated the interaction of CDF6 with GI and ZTL/FKF1/LKP2 proteins as well as its function. Yeast‐2‐hybrid (Y2H) assay experiments confirmed the interaction of full‐length, N‐terminal, and C‐terminal regions of GI with CDF6, as well as interaction of CDF6 with FKF1, ZTL, and LKP2 (Fig. 4B). *CDF6* transcript abundance was tested in plants transferred to constant light, revealing circadian regulation with a sharp peak around subjective dawn (Fig. 4C), similar to *CDF1* transcript abundance 34, 67 and the profile of CDF6 in the GI‐3F6H time series (Fig. 4A).

Since GI interacts with FKF1 and most likely CDF3 31, 35 (Fig. 3A), and because the CDF6 amino acid sequence shows high similarity to other CDFs, we predicted that CDF6 also has a similar function to other CDFs. To assess our hypothesis, transgenic plant lines were generated, in which *CDF6* was expressed from the *SUCROSE‐PROTON‐SYMPORTER* (*SUC2*) promoter that is active in phloem companion cells 36. We chose the *SUC2* promoter to drive *CDF6*, because other CDFs as well as likely target genes of CDF6 – *CO* and *FT* – are specifically expressed in phloem companion cells 72, 73. Two lines, *SUC2:HA‐CDF6* #8 and #11, accumulated higher levels of the *CDF6* transcript at ZT4 and in later time points of a qPCR time series (Fig. 4E), fluctuating around 20–60% of the WT peak level, whereas *CDF6* levels in WT were very low except at the ZT1 peak. Both transgenic lines flowered significantly later than WT under long photoperiods, with less effect under short photoperiods (Fig. 4D). If CDF6 acts in a similar way to CDF1 36, 71, we would expect it to inhibit transcription of both *CO* and *FT*. Indeed, *CO* mRNA levels were reduced at 10, 13 h and at night in the transgenic plants compared to WT, and *FT* expression was reduced more than 10‐fold at 4 h and at later time points (Fig. 4E). These results are consistent with CDF6 participating in the photoperiodic regulation of flowering, where CDF6 protein levels in WT are regulated through interaction with GI and its interacting F‐box proteins.

## Discussion

Proteostasis, the set of protein‐metabolic processes, is expected to be critical for diel rhythms in general, because the removal of transcriptional repressor proteins controls the slow timing of circadian feedback circuits 8, 9. GI indirectly mediates the degradation of transcriptional repressors through interacting with F‐box proteins involved in protein ubiquitination: ZTL mediates targeted degradation of TOC1 20 and PRR5 19 with LKP2 and FKF1 contributing 22. FKF1 targets CDF1 for degradation to regulate photoperiodic flowering, and this FKF1‐dependent degradation requires functional GI 31. GI also has protein chaperone functions to stabilize ZTL and potentially other proteins 45, 46. Our studies identified further proteostatic proteins associated with GI, and suggested links to metabolic sensing, providing candidates for the unknown targets of GI's proteostatic functions 46 and recalling previous data linking GI, metabolic inputs, and biological timing.

Overexpression of tagged *GI* (*GI‐3F6H*) under the 35S promoter in the *gi‐2* mutant background rescued the mRNA expression of *CO* and *FT* and flowering time phenotypes of the mutant. GI protein tended to greater abundance during the day than during the night, in line with its light‐dependent stabilization by ZTL 23, with an evening peak time similar to native GI protein 27. The observed immunoprecipitated protein profile closely matched the prediction of a mechanistic clock model that was informed by diverse literature data 5, indicating that the GI‐3F6H protein conformed to the dynamic, light‐responsive behavior expected from previous results (Fig. 3C).

### Confirmation of known interactors and the new direct interactor CDF6

The detection of known, indirect interactors of GI such as CUL1/2 among the weakly enriched proteins but not in the qualitative studies validated the time series approach. Among the known, direct interactors of GI that were not detected in our studies, SVP, TEM1, and TEM2 32, COP1 and ELF3 29, ELF4 24, CO 49, and TCP4 33 are observed or expected to be largely or exclusively nuclear, while SPY 74 and SOS2 40 are partly nuclear‐localized. Analysis of nuclear preparations may be necessary to enrich for these and other, nuclear interactors. Rapid, whole‐cell extraction was employed here to facilitate handling the larger sample numbers required to conduct the time series study in quintuplicate 56. Transcriptional regulators were nevertheless detected, including the known interactor CDF3 34 and its homolog CDF6 (AT1G26790). Y2H assays validated the interaction of CDF6 with GI N‐ and C‐terminal fragments, as well as with ZTL, FKF1, and LKP2 35. Functional overlap with other CDFs was confirmed, as *CDF6* overexpression in leaf phloem companion cells inhibited *CO* and *FT* transcription and delayed flowering in a photoperiod‐dependent manner (Fig. 4D, E).

*CDF6* transcript expression in long days and constant light peaks around dawn, similar to *CDF1, CDF2, CDF3,* and *CDF5* 34, 35, 67. CDF6 interaction with GI was in antiphase to FKF1 interaction, consistent with CDF6 being largely or specifically degraded *via* this F‐box protein. Our qualitative study and others conducted when GI normally accumulates 48 coincide with peak FKF1 abundance, so would not have detected CDF6 or perhaps CDF3 (Figs 3A and 4A), confirming the utility of the time series approach. However, only 10 proteins (11%) were enriched with a rhythmic profile, so the strong rhythms of FKF1 and the CDFs were uncommon. Rhythmic transcription of GI might normally confer rhythmicity on other partner proteins as it does for ZTL 18, 23, in which case we expect mis‐expression of GI to alter partner protein accumulation, as GI‐3F6H does to ZTL. Alternatively, many partner proteins might lack strong rhythmicity.

The large size and proposed proteostasis functions of GI (discussed below) risk false‐positive results. GI has not been found localized in or associated with the chloroplast but rather in the nucleus or cytoplasm 15, 23, 75. The abundant, plastid‐localized proteins enriched as interactors (Data S3 and S4) likely reflect unspecific binding, at least in the case of chloroplast‐encoded proteins, which was an expected cost of detecting low‐abundance and indirect interactors. Conservatively, we excluded mitochondrial and chloroplast proteins (see Materials and methods; Data S3) from the candidate interactors (Tables 1 and 3). GI might in principle have a physiological role in the metabolism of proteins translated on cytosolic ribosomes, prior to compartmentalization, or of proteins translocated from other compartments to the cytosol for degradation 76.

### Metabolic and nuclear functions of GI

Functionally at least, GI links carbon metabolism and timing, *via* a long‐term response of the circadian clock to sucrose 39 and the photoperiodic adjustment of the rate of starch biosynthesis 38. The trehalose‐6‐phosphate pathway mediates several such sugar responses 77, 78. *TPS8* is a paralogue without known enzymatic activity but with diurnally regulated expression, repressed by sucrose 79. GI interaction with TPS8 was highly enriched and, unusually, peaked at ZT19 (Fig. 3B), providing one of several possible mechanisms for GI to mediate between metabolism and biological timing.

Few candidate interactors were shared with a previous study using ELF3 and ELF4 bait proteins 48, which each interact with GI 24, 29. For example, RACK1A (AT1G18080) is a promiscuously interacting protein with several reported physiological roles in plants 80. Its homolog RACK1B (AT1G48630) was also weakly enriched (Table 3). Mammalian RACK1 affects the circadian clock through the interacting core clock transcription factor BMAL1 81, and contributes to degradation of its paralogue hypoxia‐induced factor HIF1a. HIF1a protein regulation is mediated *via* HSP90 and UBP (reviewed in 82): their *Arabidopsis* homologs were highly enriched in our GI‐3F6H datasets.

### Potential function of GI in cold response

Some of our candidate interactors may be used to speculate on new mechanisms contributing to GI function. GI enhances cold tolerance independently of CBF signaling 43. Two of our candidate interactors, REI1‐LIKE and GENERAL CONTROL NON‐REPRESSIBLE (GCN1; Table 3), have been implicated in cold tolerance through a role in ribosome maturation and regulation of translation initiation, respectively 83, 84. Knowledge of these potential interactors may therefore be helpful to generate hypotheses on how GI mediates cold tolerance.

### GI candidate interactors involved in protein metabolism

Protein degradation of clock‐relevant, transcriptional repressors was the first biochemical function supported for GI, acting as a scaffold for F‐box proteins, though GI's co‐chaperone function is now also implicated 18, 46. No further F‐box proteins or other ubiquitin E3 ligases were identified here, suggesting that GI mediates further physiological roles through different biochemical mechanisms. Chaperone proteins are typical, nonspecific contaminants of affinity purification studies but direct, physiologically relevant binding of HSP90 with GI has been demonstrated 46. HSP90 isoform(s) were highly enriched and weakly rhythmic in our time series (Fig. 3B). TPR4, which encodes a tetratricopeptide repeat (TPR) protein with potential to interact with HSP90/HSP70 as a co‐chaperone 85 was also strongly enriched (Table 3, Fig. 3B). The HSP70 family proteins that might function with GI and HSP90 46 were below the significance threshold in the time series study (Table 3) but one (AT5G02500) was the fourth most enriched protein in the qualitative study (Table 1).

In contrast, several other proteins involved in proteostasis were highly and reproducibly enriched. For example, in our time series, two proteasome regulatory proteins were enriched, RPT1A (AT1G53750) and RPN10 (AT4G38630) (Table 3). GI‐TAP had identified a different proteasome regulatory protein in rice 86. TCP‐1/cpn60 chaperonin family proteins (AT3G03960; AT3G20050) that can facilitate intercellular trafficking of transcription factors 87 were detected in both the time series and the qualitative studies (Tables 1 and 3).

Interestingly, a GI TAP‐MS study in rice identified a potential GI interactor whose closest *Arabidopsis* homologs, ADL3 and ADL6, are also involved in post‐Golgi vesicle trafficking 86, 88. Our purification enriched several proteins involved in trans‐Golgi or early endosome vesicle trafficking: RAB‐A2B and/or RABA3 89, TUF 90, and HAP13 91, 92. While we are not aware of any evidence for a Golgi/endosome related function of GI, these candidates may help to generate hypotheses on mechanisms of GI's to date unexplained functions. For example, *Arabidopsis* plants deficient in the Golgi‐localized transporter protein PAR1 are more resistant to paraquat due to reduced plastid accumulation of the herbicide 93, and a role of GI in stabilizing such intracellular transport proteins could be an explanation for the increased paraquat resistance of *gi* mutants in addition to the suggested increased resistance to oxidative stress 42.

UBP12 and UBP13 were highly enriched in the time series and were also detected in the preliminary and/or qualitative studies (see note added in proof). Their de‐ubiquitination activity potentially counteracts protein degradation, for example of *Arabidopsis* MYC2 94, or monoubiquitination, for example of histone H2A 95. UBP12 and UBP13 are already known to affect the *Arabidopsis* circadian clock, act upstream of *GI* and *CO* in the same photoperiodic flowering time pathway 68, and are recruited to chromatin in association with the histone methylation complex PRC2 95. In a final connection, histone de‐ubiquitination by USP7, the Drosophila homolog of UBP12/UBP13, is allosterically controlled by its interaction with a GMP synthetase 96. An *Arabidopsis* homolog (AT1G63660) of this enzyme was also enriched in the time series data (Table 3).

Our time series GI TAP‐MS results not only identified a new member of CDF proteins functioning in the photoperiodic flowering pathway but also highlighted an extended set of proteostatic functions of GI, with intriguing potential links to metabolic enzymes that are now of interest in other organisms 97. These provide a novel set of hypotheses on the biochemical mechanisms of flowering regulation and of further physiological effects of GI.

## Author contributions

Conceptualization, JK, AK, GSG, TI, AJM; Methodology, JK, YHS, RSJ, MJM, TLB; Software, JK, TLB; Formal Analysis, JK, GSG, AK, AZ, RSJ, AJM; Investigation, JK, GSG, AK; Data Curation, JK, TLB.; Writing, JK, GSG, AK, TI, AJM; Visualization, JK., GSG, AK, AJM; Supervision, KJH, TI, AJM; Funding Acquisition, JK, YHS, MJM, KJH, TLB, TI, AJM.

## Note added in proof

During preparation of this manuscript, UBP12 and UBP13 were independently identified as interactors of ZTL and LKP2 98, suggesting that these UBP proteins are indirect interactors of GI.

## Supporting information


**Fig. S1**. Validation of the GI‐TAP procedure.
**Fig. S2**. Outlier analysis of the GI‐TAP time series study.
**Fig. S3**. Transcript expression profiles of *GI* (A), *FKF1* (B), *ZTL* (C), and *FKF1* (D) from the diurnal website (http://diurnal.mocklerlab.org, [94]), using the ‘shortdays’ condition.
**Table S1**. Primer sequences.
**Data S1**. List of proteins identified by LC‐MS analysis of bands excised from silver‐stained gel after GI‐TAP (Preliminary study, Fig. 1F), includes original Mascot search output files.
**Data S2**. List of proteins identified in the qualitative, on‐bead digest analysis (Qualitative study, Fig. 1F), with peptide counts for GI‐3F6H samples and WT background controls.
**Data S3**. Proteins identified in the time series study (Fig. 1F), with quantitation and statistics, put together from output generated by scripts in Data S7, and Data S8.
**Data S4**. GO analysis on time series study: TopGO analysis results of GI‐3F6H time series.
**Data S5**. PCA on time series: R script, input files, output files; all on raw abundance data.
**Data S6**. Gene ontology analysis on time series study, for Data S4.
**Data S7**. Progenesis protein data export files, and analysis with R script for statistics on time series study (for Data S3 and Table 3).
**Data S8**. JTK_CYCLE analysis of time series study.
**Data S9**. Progenesis peptide measurements output file for time series study.

## Data Availability

Research data pertaining to this article are located at figshare.com: https://dx.doi.org/10.6084/m9.figshare.741597
