# Supplementary material for: Time‐resolved interaction proteomics of the GIGANTEA protein under diurnal cycles in Arabidopsis
Source: FEBS Lett. 2018 Dec 28;593(3):319–38. doi: 10.1002/1873-3468.13311 (PMC6373471; doi:10.1002/1873-3468.13311)
Supplement: Supplementary file 1 — Fig. S1. Validation of the GI‐TAP procedure. Fig. S2. Outlier analysis of the GI‐TAP time series study. Fig. S3. Transcript expression profiles of GI (A), FKF1 (B), ZTL (C), and FKF1 (D) from the diurnal website (http://diurnal.mocklerlab.org, [94]), using the ‘shortdays’ condition. Table S1. Primer sequences. Data S1. List of proteins identified by LC‐MS analysis of bands excised from silver‐stained gel after GI‐TAP (Preliminary study, Fig. 1F), includes original Mascot search output files. Data S2. List of proteins identified in the qualitative, on‐bead digest analysis (Qualitative study, Fig. 1F), with peptide counts for GI‐3F6H samples and WT background controls. Data S3. Proteins identified in the time series study (Fig. 1F), with quantitation and statistics, put together from output generated by scripts in Data S7, and Data S8. Data S4. GO analysis on time series study: TopGO analysis results of GI‐3F6H time series. Data S5. PCA on time series: R script, input files, output files; all on raw abundance data. Data S6. Gene ontology analysis on time series study, for Data S4. Data S7. Progenesis protein data export files, and analysis with R script for statistics on time series study (for Data S3 and Table 3). Data S8. JTK_CYCLE analysis of time series study. Data S9. Progenesis peptide measurements output file for time series study. [file FEB2-593-319-s001.zip › feb213311-sup-0014-SupportingDataFiles .docx]

**Supplemental Information**

**Overview of supporting data files uploaded separately:**

| **Data file name** | **File contents** |
| --- | --- |
| **Data S1.xlsx** | List of proteins identified by LC-MS analysis of bands excised from silver-stained gel after GI-TAP (Preliminary study, Figure 1F), includes original Mascot search output files |
| **Data S2.xlsx** | List of proteins identified in the qualitative, on-bead digest analysis (Qualitative study, Figure 1F), with peptide counts for GI-3F6H samples and WT background controls |
| **Data S3.xlsx** | Proteins identified in the time series study (Figure 1F), with quantitation and statistics, put together from output generated by scripts in Data S7, and Data S8 |
| **Data S4.xlsx** | GO analysis on time series study: TopGO analysis results of GI-3F6H time series |
| **Data S5.zip** | Principal Component Analysis on time series: R script, input files, output files; all on raw abundance data |
| R_corr_PCA.R | R script for correlation analysis and PCA |
| input_corr_PCA.csv | input file for correlation analysis and PCA (Progenesis output data) |
| corr_all.pdf | correlation analysis results for all samples including the WT background, same as Figure S2 |
| PCA_all.pdf | PCA on raw data, all samples |
| PCA_GI.pdf | PCA on raw data, only GI-3F6H samples |
| PCA_GI_without19E | PCA on raw data, only GI-3F6H samples and without outlier 19E, as Figure 2F |
| **Data S6.zip** | Gene ontology analysis on time series study, for Data S4 |
| GO_GI-TAP-timecourse.R | R script implementing topGO analysis of proteins significantly enriched by GI-3F6H TAP |
| topGO_input_stats_raw.csv | stats input file for GO analysis (data taken from Data S3) |
| topGO_output_allIDs.xlsx | output file of GO analysis for all identifications |
| topGO_output_min2IDs | output file of GO analysis for proteins with more than 1 identified peptides |
| gene-association.csv | GO database used for topGO analysis |
| **Data S7.zip** | Progenesis protein data export files, and analysis with R script for statistics on time series study (for Data S3 and Table 3) |
| R_stats_plots.R | R script for statistics and plotting |
| Progenesis_protein_  measurements_raw.csv | Input file for script; Progenesis protein measurement output, raw abundance |
| Progenesis_protein_  measurements_norm.csv | Progenesis protein measurement output, normalized abundance |
| overlap_with_GFP-TAP.csv | List of proteins that were previously found to bind to GFP-3F6H [46]; list is used by script to exclude these proteins from statistical analysis |
| plastid_proteins.csv | List of proteins that are predicted to be in the plastid or mitochondria but not the nucleus or cytoplasm, by GO annotation; list is used be the script to exclude these proteins from statistical analysis |
| R_stats_output | output file of script R_stats_plots.R |
| **Data S8.zip** | JTK_CYCLE analysis of time series study |
| R_run_JTK_CYCLE.R | R script implementing JTK_CYCLE analysis on the time series protein abundance data. |
| JTK_CYCLE.R | JTK_CYCLE R file, original from [51] |
| metadata.csv | a metadata input file needed to run R_run_JTK_CYCLE |
| JTK_input.csv | input file into JTK_CYCLE analysis (Progenesis raw protein measurements) |
| JTK_output.xlsx | output file from JTK_CYCLE analysis, was integrated into Data S3 using Excel's vlookup function |
| **Data S9.csv** | Progenesis peptide measurements output file for time series study. |
