# Supplementary figures and images for: Time‐resolved interaction proteomics of the GIGANTEA protein under diurnal cycles in Arabidopsis
Source: FEBS Lett. 2018 Dec 28;593(3):319–38. doi: 10.1002/1873-3468.13311 (PMC6373471; doi:10.1002/1873-3468.13311)

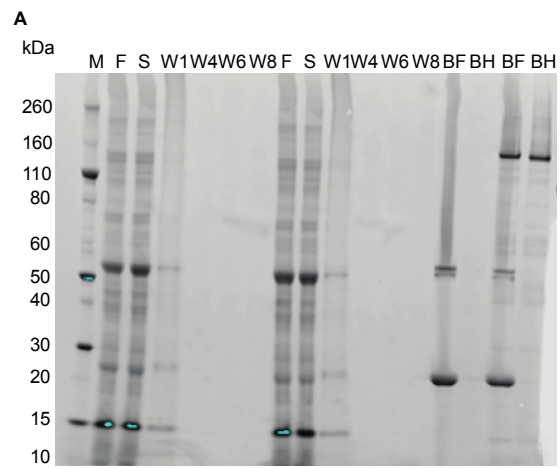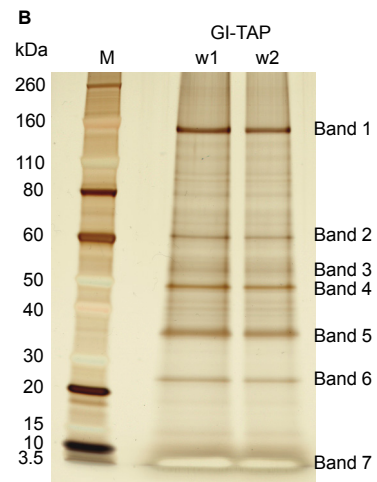

Supplement: Supplementary file 1 — Fig. S1. Validation of the GI‐TAP procedure. Fig. S2. Outlier analysis of the GI‐TAP time series study. Fig. S3. Transcript expression profiles of GI (A), FKF1 (B), ZTL (C), and FKF1 (D) from the diurnal website (http://diurnal.mocklerlab.org, [94]), using the ‘shortdays’ condition. Table S1. Primer sequences. Data S1. List of proteins identified by LC‐MS analysis of bands excised from silver‐stained gel after GI‐TAP (Preliminary study, Fig. 1F), includes original Mascot search output files. Data S2. List of proteins identified in the qualitative, on‐bead digest analysis (Qualitative study, Fig. 1F), with peptide counts for GI‐3F6H samples and WT background controls. Data S3. Proteins identified in the time series study (Fig. 1F), with quantitation and statistics, put together from output generated by scripts in Data S7, and Data S8. Data S4. GO analysis on time series study: TopGO analysis results of GI‐3F6H time series. Data S5. PCA on time series: R script, input files, output files; all on raw abundance data. Data S6. Gene ontology analysis on time series study, for Data S4. Data S7. Progenesis protein data export files, and analysis with R script for statistics on time series study (for Data S3 and Table 3). Data S8. JTK_CYCLE analysis of time series study. Data S9. Progenesis peptide measurements output file for time series study. [file FEB2-593-319-s001.zip › feb213311-sup-0001-FigS1.pdf]

A

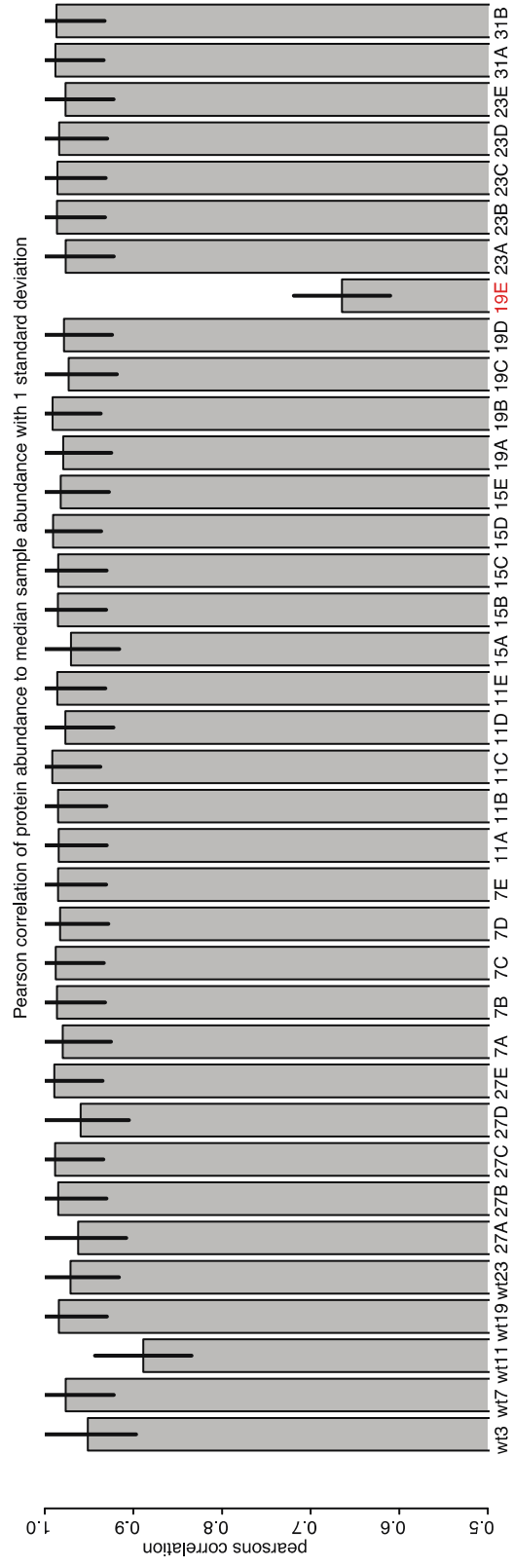

B

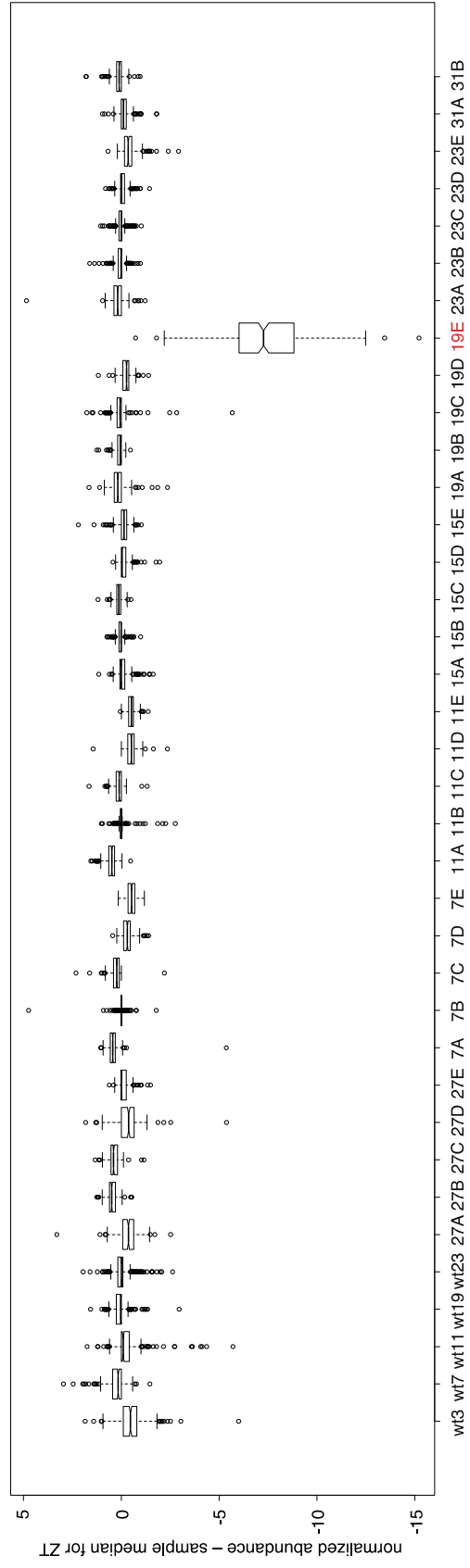

Supplement: Supplementary file 1 — Fig. S1. Validation of the GI‐TAP procedure. Fig. S2. Outlier analysis of the GI‐TAP time series study. Fig. S3. Transcript expression profiles of GI (A), FKF1 (B), ZTL (C), and FKF1 (D) from the diurnal website (http://diurnal.mocklerlab.org, [94]), using the ‘shortdays’ condition. Table S1. Primer sequences. Data S1. List of proteins identified by LC‐MS analysis of bands excised from silver‐stained gel after GI‐TAP (Preliminary study, Fig. 1F), includes original Mascot search output files. Data S2. List of proteins identified in the qualitative, on‐bead digest analysis (Qualitative study, Fig. 1F), with peptide counts for GI‐3F6H samples and WT background controls. Data S3. Proteins identified in the time series study (Fig. 1F), with quantitation and statistics, put together from output generated by scripts in Data S7, and Data S8. Data S4. GO analysis on time series study: TopGO analysis results of GI‐3F6H time series. Data S5. PCA on time series: R script, input files, output files; all on raw abundance data. Data S6. Gene ontology analysis on time series study, for Data S4. Data S7. Progenesis protein data export files, and analysis with R script for statistics on time series study (for Data S3 and Table 3). Data S8. JTK_CYCLE analysis of time series study. Data S9. Progenesis peptide measurements output file for time series study. [file FEB2-593-319-s001.zip › feb213311-sup-0002-FigS2.pdf]

A

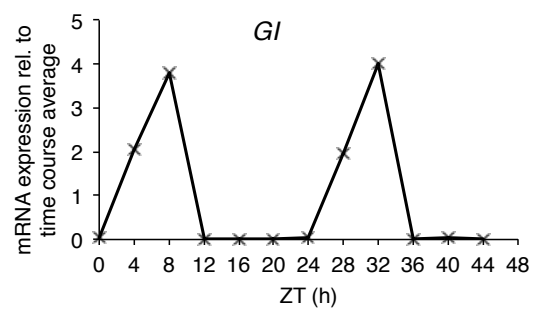

B

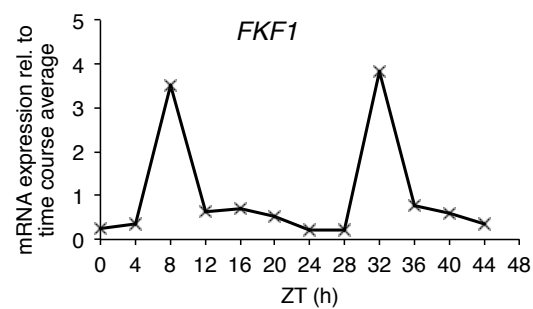

C

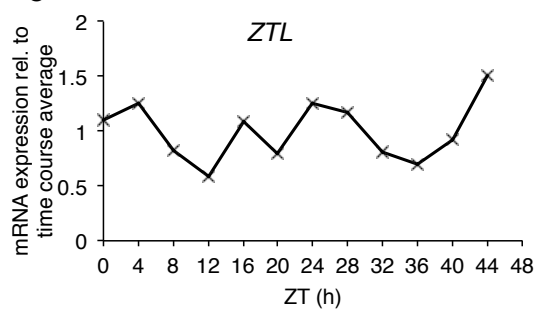

D

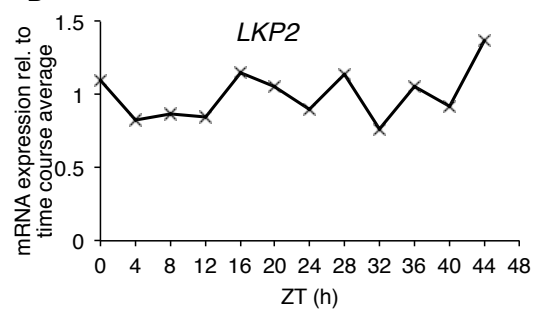

Supplement: Supplementary file 1 — Fig. S1. Validation of the GI‐TAP procedure. Fig. S2. Outlier analysis of the GI‐TAP time series study. Fig. S3. Transcript expression profiles of GI (A), FKF1 (B), ZTL (C), and FKF1 (D) from the diurnal website (http://diurnal.mocklerlab.org, [94]), using the ‘shortdays’ condition. Table S1. Primer sequences. Data S1. List of proteins identified by LC‐MS analysis of bands excised from silver‐stained gel after GI‐TAP (Preliminary study, Fig. 1F), includes original Mascot search output files. Data S2. List of proteins identified in the qualitative, on‐bead digest analysis (Qualitative study, Fig. 1F), with peptide counts for GI‐3F6H samples and WT background controls. Data S3. Proteins identified in the time series study (Fig. 1F), with quantitation and statistics, put together from output generated by scripts in Data S7, and Data S8. Data S4. GO analysis on time series study: TopGO analysis results of GI‐3F6H time series. Data S5. PCA on time series: R script, input files, output files; all on raw abundance data. Data S6. Gene ontology analysis on time series study, for Data S4. Data S7. Progenesis protein data export files, and analysis with R script for statistics on time series study (for Data S3 and Table 3). Data S8. JTK_CYCLE analysis of time series study. Data S9. Progenesis peptide measurements output file for time series study. [file FEB2-593-319-s001.zip › feb213311-sup-0003-FigS3.pdf]
