# Supplementary material for: Time‐resolved interaction proteomics of the GIGANTEA protein under diurnal cycles in Arabidopsis
Source: FEBS Lett. 2018 Dec 28;593(3):319–38. doi: 10.1002/1873-3468.13311 (PMC6373471; doi:10.1002/1873-3468.13311)
Supplement: Supplementary file 2 [file FEB2-593-319-s002.docx]

# Supporting Experimental Procedures

**Methods S1. Verification of GI:3F6H expression by Western Blotting and Silver-stain gel followed by in-gel digestion**

Western blot analysis shown in Figure S1 was performed as described (Song et al., 2014), using a commercial 4-12% Bis-Tris PAGE (Life Technologies) and the iBlot system according to the manufacturer’s instructions. The M2 mouse anti-Flag antibody (Sigma) and the IRDYE® 800 CW goat anti mouse secondary antibody (Licor) were used for detection of anti-Flag signal on the membrane using a Licor Odyssey fluorescence scanner. For silver staining, the SilverQuestTM silver staining kit (Life Technologies) was used according to the manufacturer’s instructions. Mass spectrometry results of in-gel digestion of specific bands are reported in data S1.

**Methods S2. Protein extraction and enrichment by TAP for mass spectrometric analysis**

Frozen plant tissue was ground to a fine powder in a liquid nitrogen and dry ice-cooled mortar and processed essentially as described [47]. For protein extraction, one tissue volume of SII buffer (100 mM sodium phosphate pH7.4, 150 mM KCl, 5 mM EDTA, 5 mM EGTA, 0.1% TritonX-100) or RIPA buffer (50 mM Tris pH 7.5, 150 mM KCl, 1% NP-40, 0.5% Dexoycholate) with 1 Complete protease inhibitor Cocktail EDTA free mini tablet (Roche) per 10 ml, PhosStop phosphatase inhibitor mixture (Roche), 50 µM MG-132 and 1 mM PMSF was added to the tissue and tubes were vortexed vigorously. The two different buffers were used in the qualitative study to test whether one may perform better than the other but since the results were comparable only SII was used for the time series study. Crude extracts were sonicated with a sonicating probe at 10 µM amplitude for 10s three times, cleared at 3220xg twice and filtered through 0.45 µm syringe filters. Protein was quantified with a standard Bradford assay. The remaining TAP procedures and mass spectrometry were as described [47].

For the time series, extract containing 28 mg of protein was used for TAP. For AP on anti-FLAG M2 magnetic beads, a ratio of 10 µl of beads (20 µl of supplied 50% slurry, Sigma) per 4 mg of protein was used. To equilibrate, beads were washed in ten packed gel volumes of TBS (50 mM Tris HCl, 150 mM NaCl, pH 7.4) twice, followed by one wash with SII or RIPA buffer without inhibitors. Beads were added to appropriate volume of extract as a 50% slurry and the mix was incubated on a rotating wheel at 4°C for 2h for binding of protein to the beads. Beads were washed twice with 20 bead volumes and once with ten bead volumes of SII buffer without inhibitors (1 bead volume being 100% bead gel), followed by two washes with 10 bead volumes of Flag-to-His buffer (0.5 M NaPhosphate pH7.4, 150 mM KCl, 0.05% TritonX-100). Protein was eluted 3 times with 2 bead volumes each of Flag-to-His buffer containing 250 µg/ml 3xFLAG peptide (sigma) for 15 min each. For 6xHis tag purification, 50 µl of bead slurry were used for each sample. Dynabeads® for His purification (Life Technologies; time series) or Dyna1 Protein G magnetic beads (qualitative study) were equilibrated by washing with 20 slurry volumes of Flag to His buffer twice. Combined eluates were incubated with the beads on a rotating wheel for 15 min. Beads were washed twice with 1 ml of Flag-to-His buffer followed by three washes with 1 ml of freshly prepared 25 mM Ammonium Bicarbonate (Sigma). After removal of the final wash, beads were stored at -80°C until on-bead digest.

**Methods S3. Protein digestion, peptide and mass spectrometric analysis**

For the qualitative study, the protein digestion, peptide analysis and mass spectrometry were as described [47].

For the time series, on-bead digest was performed at room temperature as follows: 25 µl 10 mM DTT was added to the beads and samples were incubated at room temperature for 30min to reduce sulfhydryl groups. For carbamidomethylation, 50 µl of 25 mM iodoacetamide (sigma) were added and samples were incubated in the dark for 1 h. 25 µl of 8 M urea, 2.5 µl of 1 M ABC and 1.25 µl of 1 g/l trypsin (Worthington) were added and protein was digested overnight. An additional 0.25 µl of 1 g/l trypsin was added and digestion was performed for an additional 6 h with occasional vortexing.

Peptides in the digest solution were separated from the Dynabeads® and desalted on a reverse phase resin using BondElute columns (25MG columns, Agilent): two times 1 ml methanol and two times 1 ml HPLC grade water (Thermo Fisher) were sequentially passed through the column before loading the sample. After binding of peptides, columns were washed using 1 ml of HPLC grade water and peptides were eluted in 1 ml acetonitrile. Peptides were vacuum dried in a Speed-vac (RC1010, Thermo).

Dried peptides were dissolved in 8 µl 0.05% TFA and passed through Millex-LH

0.45 µm (Millipore) filters. 5 µl were analysed by mass spectrometry. Nano-HPLC- MS/MS analysis was performed using an on-line system consisting of a nano-pump (Dionex Ultimate 3000, Thermo-Fisher, UK) coupled to a QExactive instrument (Thermo-Fisher, UK) with a pre-column of 300 µm x 5 mm (Acclaim Pepmap, 5 µm particle size) connected to a column of 75 µm x 50 cm (Acclaim Pepmap, 3 µm particle size). Samples were analyzed on a 90min gradient in data dependent analysis (1 survey scan at 70k resolution followed by the top 5 MS/MS). One replicate of each time point was analyzed in random order before the next set of replicates of all time points, to rule out effects of instrument drift on quantitation.
