# Supplementary material for: Time‐resolved interaction proteomics of the GIGANTEA protein under diurnal cycles in Arabidopsis
Source: FEBS Lett. 2018 Dec 28;593(3):319–38. doi: 10.1002/1873-3468.13311 (PMC6373471; doi:10.1002/1873-3468.13311)
Supplement: Supplementary file 1 — Fig. S1. Validation of the GI‐TAP procedure. Fig. S2. Outlier analysis of the GI‐TAP time series study. Fig. S3. Transcript expression profiles of GI (A), FKF1 (B), ZTL (C), and FKF1 (D) from the diurnal website (http://diurnal.mocklerlab.org, [94]), using the ‘shortdays’ condition. Table S1. Primer sequences. Data S1. List of proteins identified by LC‐MS analysis of bands excised from silver‐stained gel after GI‐TAP (Preliminary study, Fig. 1F), includes original Mascot search output files. Data S2. List of proteins identified in the qualitative, on‐bead digest analysis (Qualitative study, Fig. 1F), with peptide counts for GI‐3F6H samples and WT background controls. Data S3. Proteins identified in the time series study (Fig. 1F), with quantitation and statistics, put together from output generated by scripts in Data S7, and Data S8. Data S4. GO analysis on time series study: TopGO analysis results of GI‐3F6H time series. Data S5. PCA on time series: R script, input files, output files; all on raw abundance data. Data S6. Gene ontology analysis on time series study, for Data S4. Data S7. Progenesis protein data export files, and analysis with R script for statistics on time series study (for Data S3 and Table 3). Data S8. JTK_CYCLE analysis of time series study. Data S9. Progenesis peptide measurements output file for time series study. [file FEB2-593-319-s001.zip › feb213311-sup-0009-DataS5.zip!/corr_all.pdf]

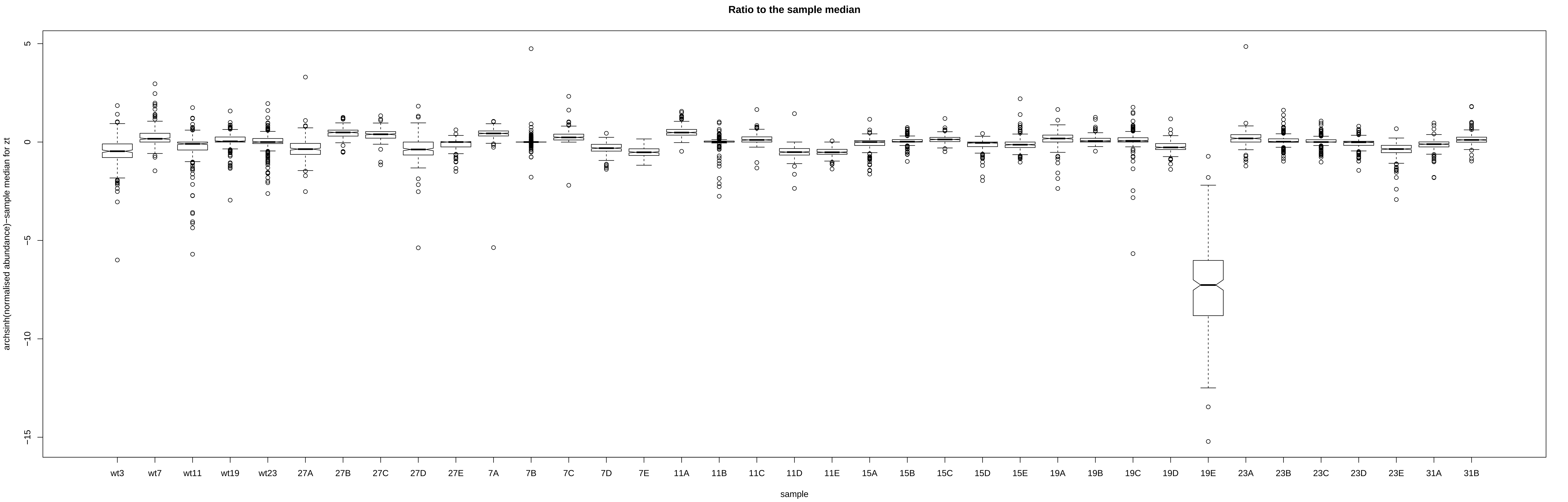
Ratio to the sample median
archsinh(normalised abundance)−sample median for zt
sample
5
0
−5
−10
−15
wt3
wt7
wt11
wt19
wt23
27A
27B
27C
27D
27E
7A
7B
7C
7D
7E
11A
11B
11C
11D
11E
15A
15B
15C
15D
15E
19A
19B
19C
19D
19E
23A
23B
23C
23D
23E
31A
31B

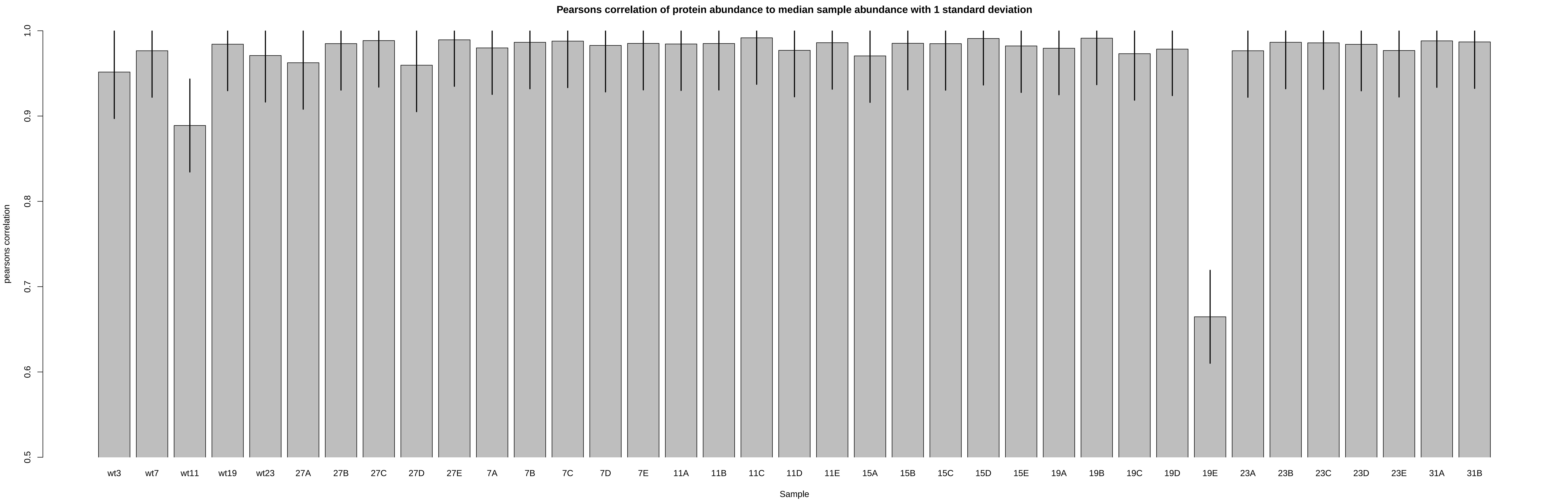
Pearsons correlation of protein abundance to median sample abundance with 1 standard deviation
pearsons correlation
0.5
0.6
0.7
0.8
0.9
1.0
wt3
wt7
wt11
wt19
wt23
27A
27B
27C
27D
27E
7A
7B
7C
7D
7E
11A
11B
11C
11D
11E
15A
15B
15C
15D
15E
19A
19B
19C
19D
19E
23A
23B
23C
23D
23E
31A
31B
Sample
