# Supplementary material for: Time‐resolved interaction proteomics of the GIGANTEA protein under diurnal cycles in Arabidopsis
Source: FEBS Lett. 2018 Dec 28;593(3):319–38. doi: 10.1002/1873-3468.13311 (PMC6373471; doi:10.1002/1873-3468.13311)
Supplement: Supplementary file 1 — Fig. S1. Validation of the GI‐TAP procedure. Fig. S2. Outlier analysis of the GI‐TAP time series study. Fig. S3. Transcript expression profiles of GI (A), FKF1 (B), ZTL (C), and FKF1 (D) from the diurnal website (http://diurnal.mocklerlab.org, [94]), using the ‘shortdays’ condition. Table S1. Primer sequences. Data S1. List of proteins identified by LC‐MS analysis of bands excised from silver‐stained gel after GI‐TAP (Preliminary study, Fig. 1F), includes original Mascot search output files. Data S2. List of proteins identified in the qualitative, on‐bead digest analysis (Qualitative study, Fig. 1F), with peptide counts for GI‐3F6H samples and WT background controls. Data S3. Proteins identified in the time series study (Fig. 1F), with quantitation and statistics, put together from output generated by scripts in Data S7, and Data S8. Data S4. GO analysis on time series study: TopGO analysis results of GI‐3F6H time series. Data S5. PCA on time series: R script, input files, output files; all on raw abundance data. Data S6. Gene ontology analysis on time series study, for Data S4. Data S7. Progenesis protein data export files, and analysis with R script for statistics on time series study (for Data S3 and Table 3). Data S8. JTK_CYCLE analysis of time series study. Data S9. Progenesis peptide measurements output file for time series study. [file FEB2-593-319-s001.zip › feb213311-sup-0009-DataS5.zip!/PCA_all.pdf]

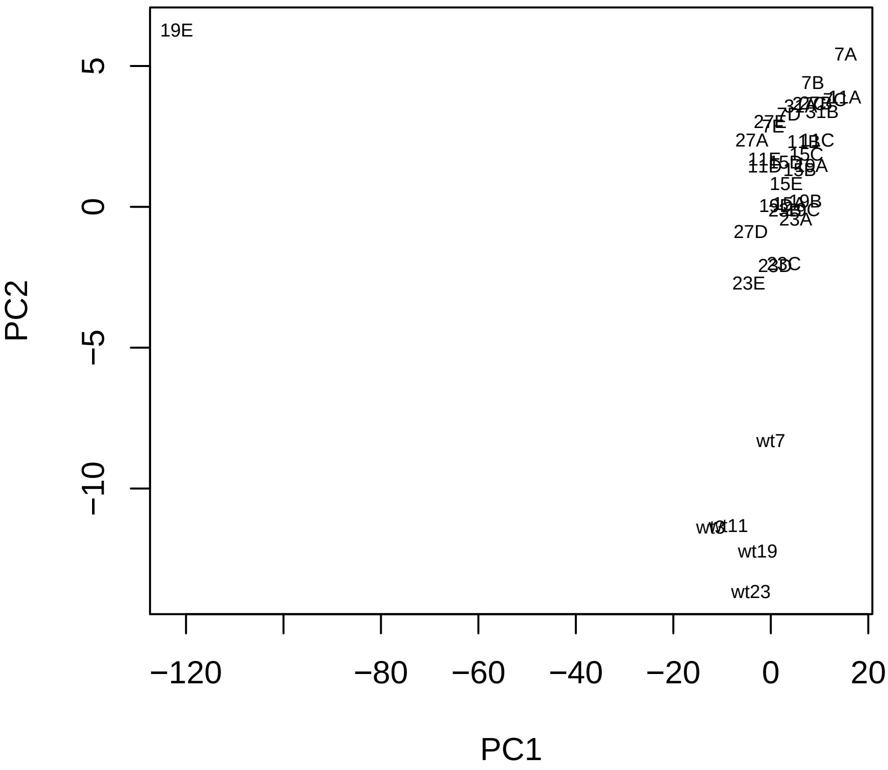
PC1
PC2
−120
−80
−60
−40
−20
0
20
5
0
−5
−10
19E
7A
7B
11A
31B
27E
7E
7D
27A
11C
15C
11D
15E
19B
23A
27D
23C
23D
23E
wt7
wt11
wt19
wt23

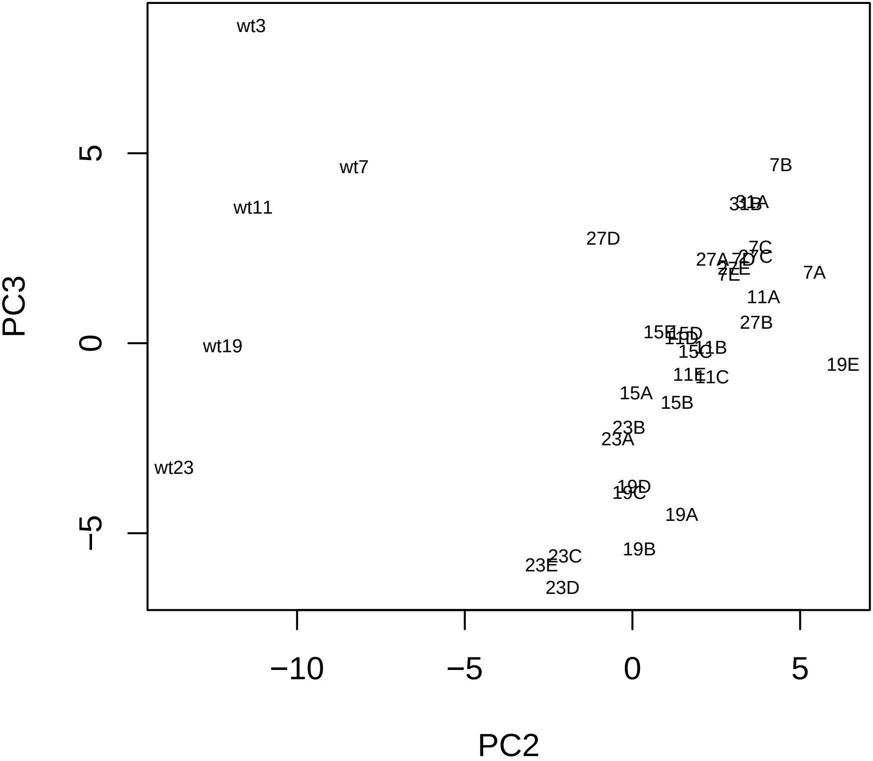

wt3
wt7
wt11
wt19
wt23
7B
27D
7A
11A
27B
19E
15A
15B
23B
23A
19A
19B
23C
23E
23D
11C
5
0
−5
−10
−5
0
5
PC3
PC2

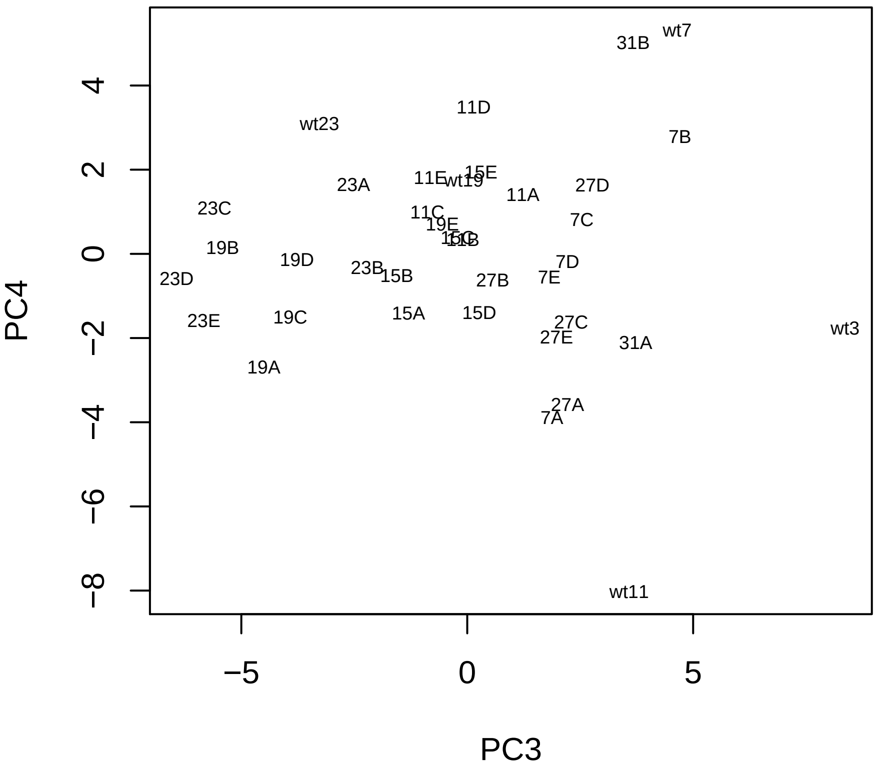

PC4
PC3
4
2
0
−2
−4
−6
−8
−5
0
5
wt7
31B
11D
wt23
7B
15E
11E
wt19
23A
27D
11A
23C
11C
7C
19E
15C
11B
19B
19D
7D
23B
15B
23D
27B
7E
15A
15D
19C
23E
27C
wt3
27E
31A
19A
27A
7A
wt11

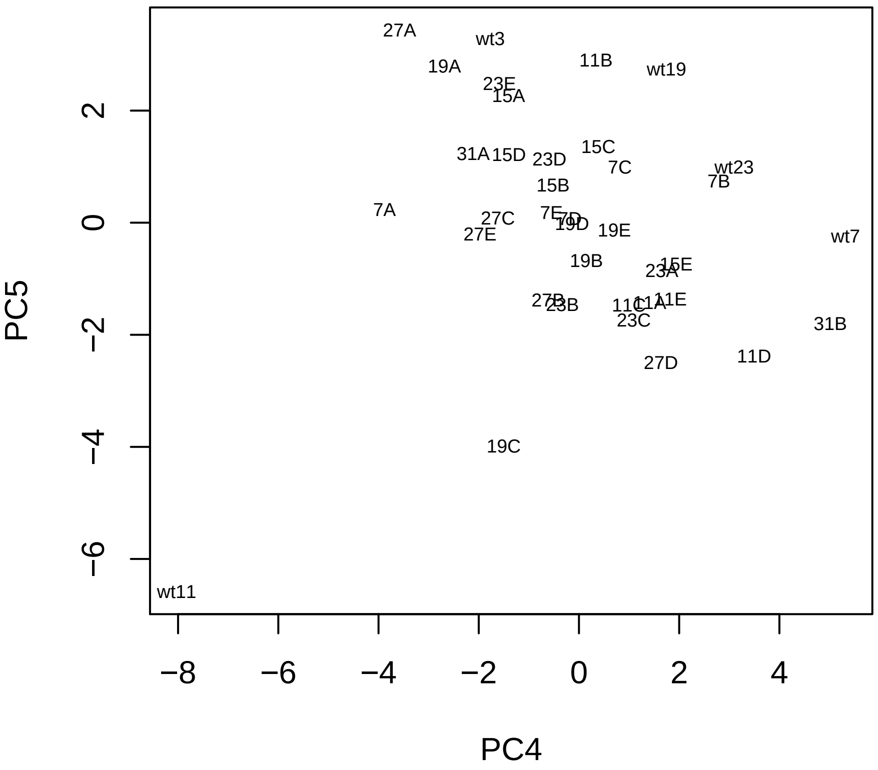

27A
wt3
19A
11B
wt19
23E
15A
31A
15D
23D
15C
7C
wt23
7B
15B
7A
27C
7E
7D
19D
19E
27E
wt7
19B
15E
23A
27B
23B
11C
11A
11E
23C
31B
27D
11D
19C
wt11
−8
−6
−4
−2
0
2
4
PC4
PC5

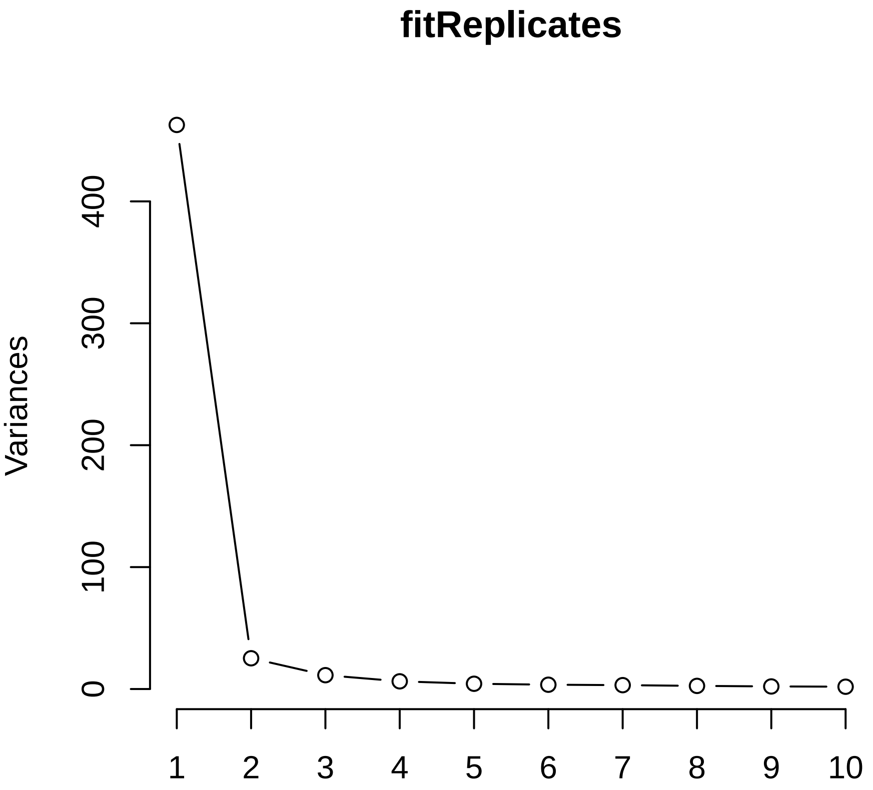
fitReplicates
Variances
0
100
200
300
400
1
2
3
4
5
6
7
8
9
10
