# Supplementary material for: Time‐resolved interaction proteomics of the GIGANTEA protein under diurnal cycles in Arabidopsis
Source: FEBS Lett. 2018 Dec 28;593(3):319–38. doi: 10.1002/1873-3468.13311 (PMC6373471; doi:10.1002/1873-3468.13311)
Supplement: Supplementary file 1 — Fig. S1. Validation of the GI‐TAP procedure. Fig. S2. Outlier analysis of the GI‐TAP time series study. Fig. S3. Transcript expression profiles of GI (A), FKF1 (B), ZTL (C), and FKF1 (D) from the diurnal website (http://diurnal.mocklerlab.org, [94]), using the ‘shortdays’ condition. Table S1. Primer sequences. Data S1. List of proteins identified by LC‐MS analysis of bands excised from silver‐stained gel after GI‐TAP (Preliminary study, Fig. 1F), includes original Mascot search output files. Data S2. List of proteins identified in the qualitative, on‐bead digest analysis (Qualitative study, Fig. 1F), with peptide counts for GI‐3F6H samples and WT background controls. Data S3. Proteins identified in the time series study (Fig. 1F), with quantitation and statistics, put together from output generated by scripts in Data S7, and Data S8. Data S4. GO analysis on time series study: TopGO analysis results of GI‐3F6H time series. Data S5. PCA on time series: R script, input files, output files; all on raw abundance data. Data S6. Gene ontology analysis on time series study, for Data S4. Data S7. Progenesis protein data export files, and analysis with R script for statistics on time series study (for Data S3 and Table 3). Data S8. JTK_CYCLE analysis of time series study. Data S9. Progenesis peptide measurements output file for time series study. [file FEB2-593-319-s001.zip › feb213311-sup-0009-DataS5.zip!/PCA_GI.pdf]

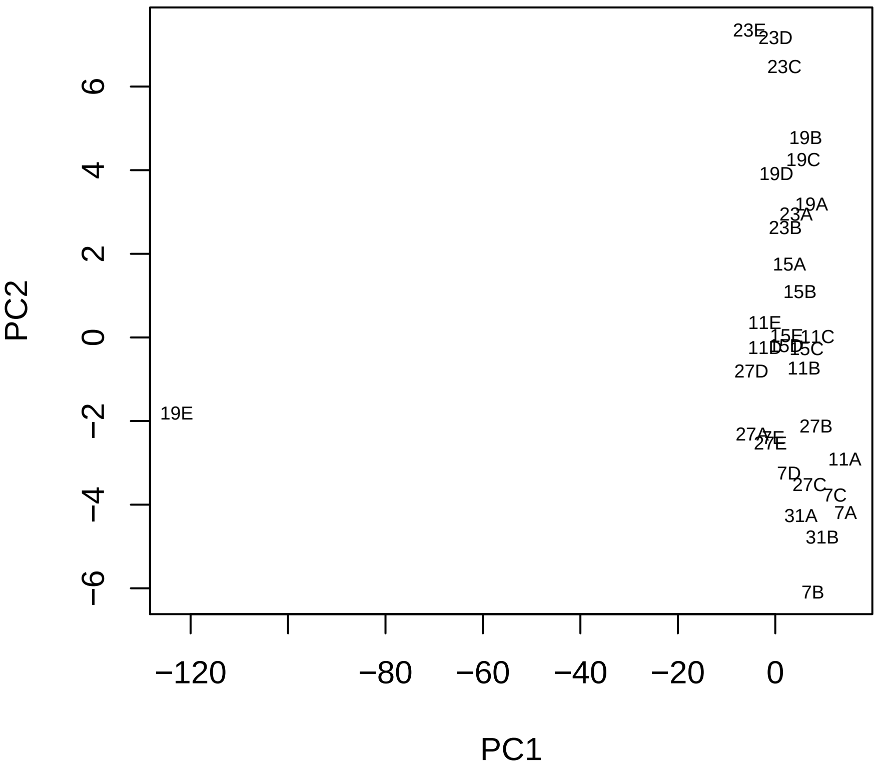

PC1
PC2
−120
−80
−60
−40
−20
0
−6
−4
−2
0
2
4
6
23E
23D
23C
19B
19C
19D
19A
23A
23B
15A
15B
11E
15E
11C
11D
15D
15C
27D
11B
19E
27B
27A
7E
27E
11A
7D
27C
7C
31A
7A
31B
7B

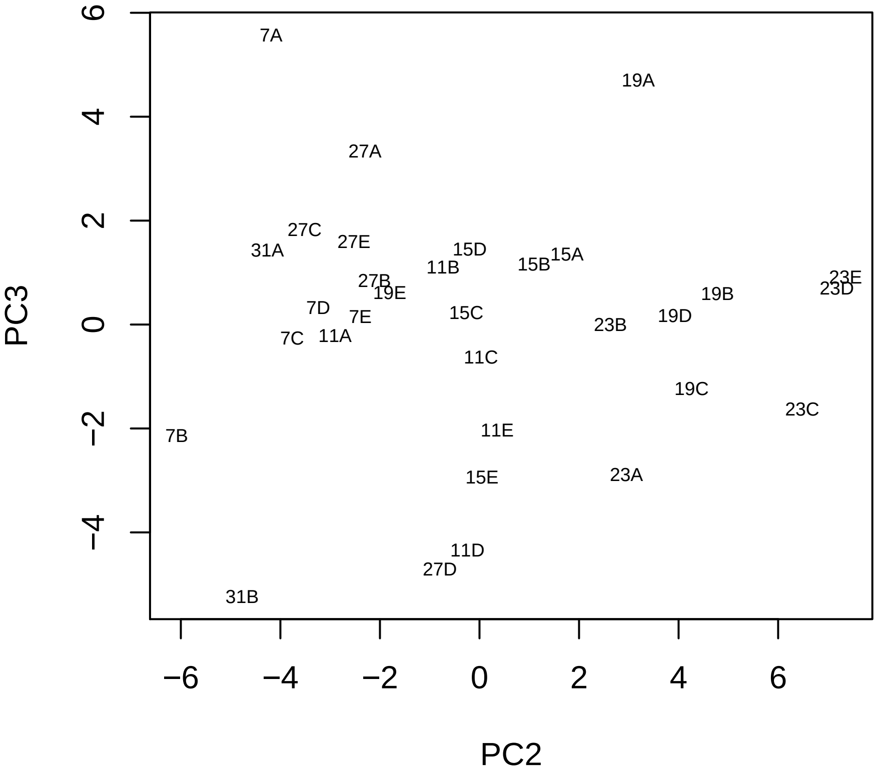
PC3
PC2
6
4
2
0
−2
−4
−6
−4
−2
0
2
4
6
7A
19A
27A
27C
31A
27E
15D
15A
15B
11B
23E
23D
27B
19E
19B
7D
7E
15C
19D
23B
7C
11A
11C
19C
23C
7B
11E
15E
23A
11D
27D
31B

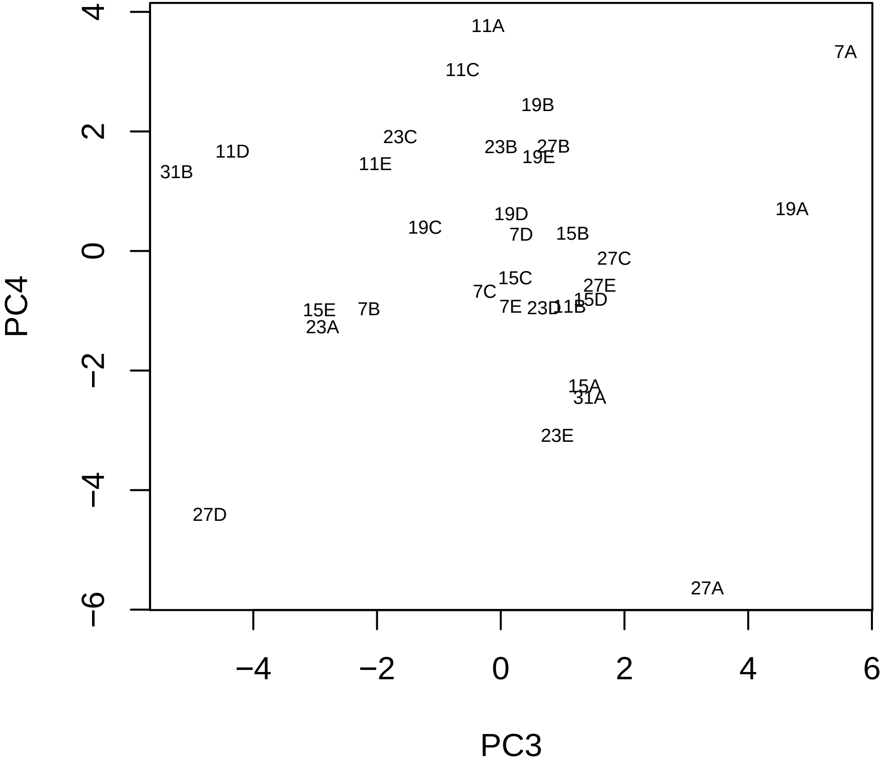

PC4
PC3
4
2
0
−2
−4
−6
−4
−2
0
2
4
6
11A
7A
11C
19B
23C
11D
23B
27B
19E
11E
31B
19A
19D
19C
7D
15B
27C
15C
27E
7C
15D
7E
23D
11B
15E
7B
23A
15A
31A
23E
27D
27A

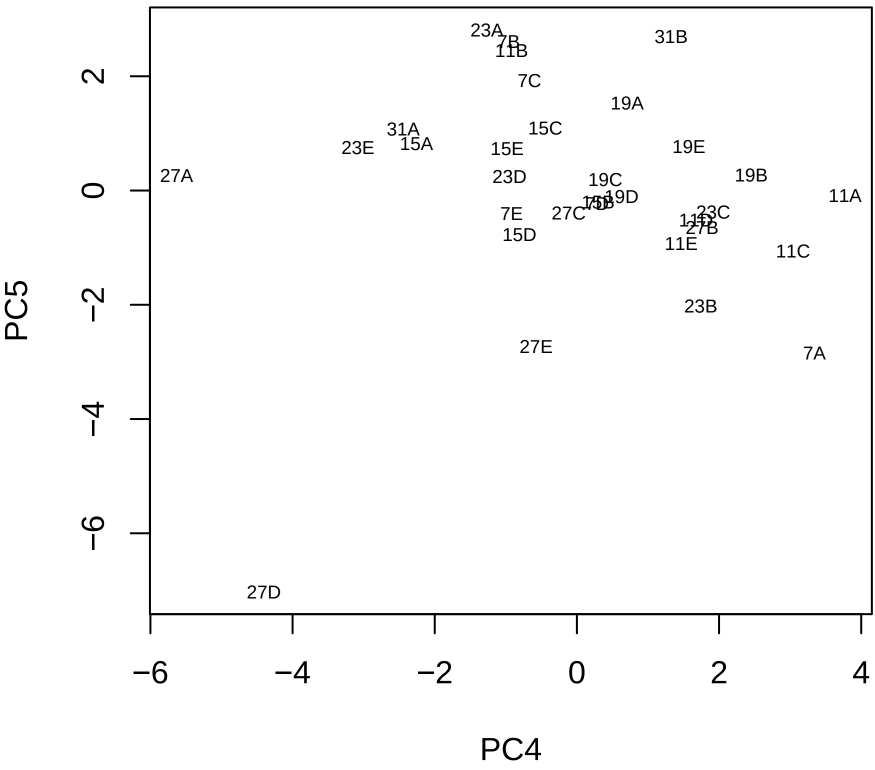

PC5
PC4
2
0
−2
−4
−6
−6
−4
−2
0
2
4
23A
7B
11B
31B
7C
19A
31A
15C
23E
15A
15E
19E
27A
23D
19C
19B
15B
7D
19D
11A
7E
27C
23C
11D
27B
15D
11E
11C
23B
27E
7A
27D

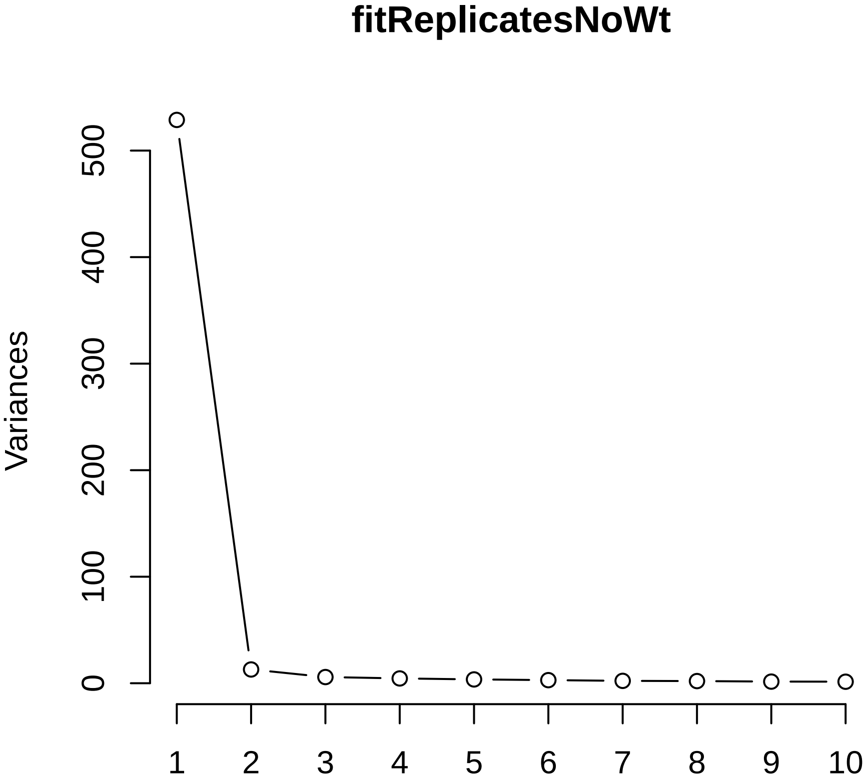
fitReplicatesNoWt
Variances
0
100
200
300
400
500
1
2
3
4
5
6
7
8
9
10
