# Supplementary material for: Time‐resolved interaction proteomics of the GIGANTEA protein under diurnal cycles in Arabidopsis
Source: FEBS Lett. 2018 Dec 28;593(3):319–38. doi: 10.1002/1873-3468.13311 (PMC6373471; doi:10.1002/1873-3468.13311)
Supplement: Supplementary file 1 — Fig. S1. Validation of the GI‐TAP procedure. Fig. S2. Outlier analysis of the GI‐TAP time series study. Fig. S3. Transcript expression profiles of GI (A), FKF1 (B), ZTL (C), and FKF1 (D) from the diurnal website (http://diurnal.mocklerlab.org, [94]), using the ‘shortdays’ condition. Table S1. Primer sequences. Data S1. List of proteins identified by LC‐MS analysis of bands excised from silver‐stained gel after GI‐TAP (Preliminary study, Fig. 1F), includes original Mascot search output files. Data S2. List of proteins identified in the qualitative, on‐bead digest analysis (Qualitative study, Fig. 1F), with peptide counts for GI‐3F6H samples and WT background controls. Data S3. Proteins identified in the time series study (Fig. 1F), with quantitation and statistics, put together from output generated by scripts in Data S7, and Data S8. Data S4. GO analysis on time series study: TopGO analysis results of GI‐3F6H time series. Data S5. PCA on time series: R script, input files, output files; all on raw abundance data. Data S6. Gene ontology analysis on time series study, for Data S4. Data S7. Progenesis protein data export files, and analysis with R script for statistics on time series study (for Data S3 and Table 3). Data S8. JTK_CYCLE analysis of time series study. Data S9. Progenesis peptide measurements output file for time series study. [file FEB2-593-319-s001.zip › feb213311-sup-0009-DataS5.zip!/PCA_GI_without19E.pdf]

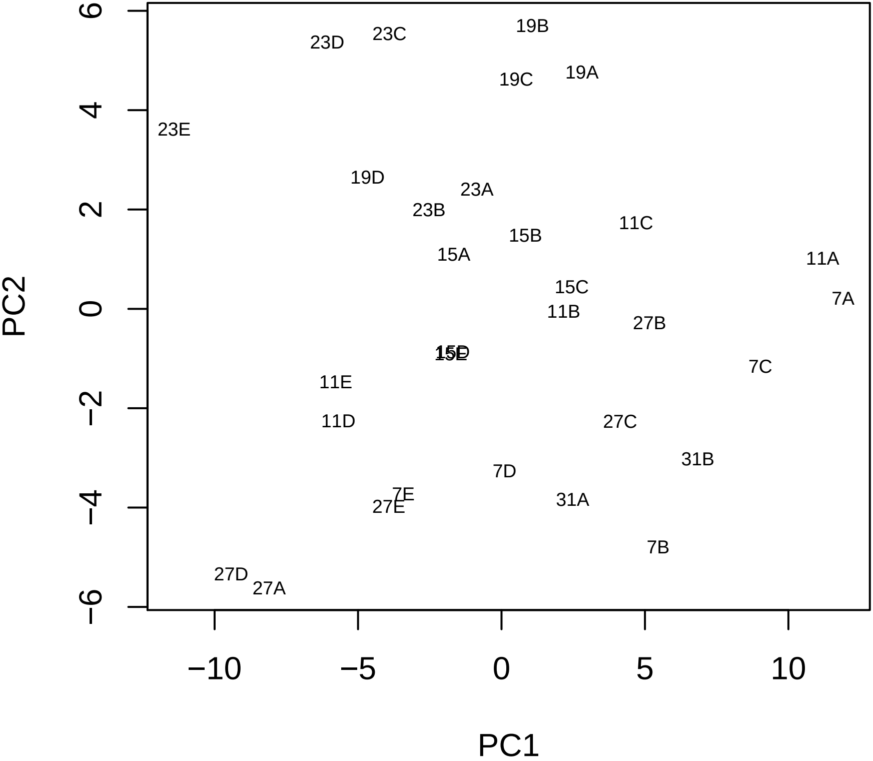
PC1
PC2
−10
−5
0
5
10
−6
−4
−2
0
2
4
6
19B
23C
23D
19A
19C
23E
19D
23A
23B
11C
15B
15A
11A
15C
7A
11B
27B
15D
15E
7C
11E
11D
27C
31B
7D
7E
27E
31A
7B
27D
27A

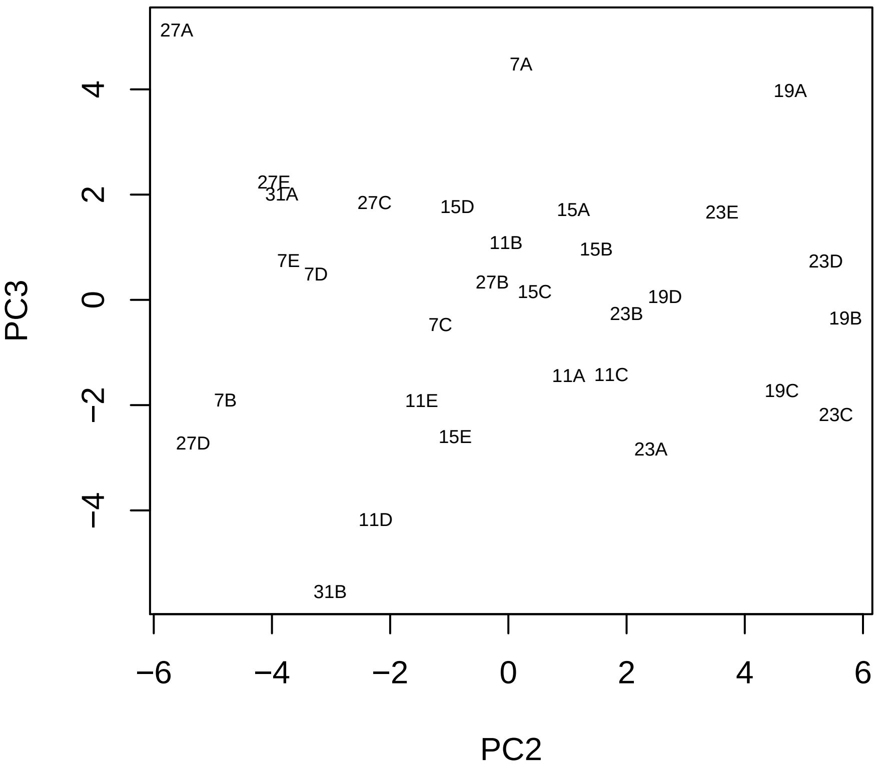

27A
7A
19A
27E
31A
27C
15D
15A
23E
11B
15B
7E
7D
23D
27B
15C
19D
23B
19B
7C
11A
11C
19C
7B
11E
23C
15E
27D
23A
11D
31B
PC3
PC2
−6
−4
−2
0
2
4
6

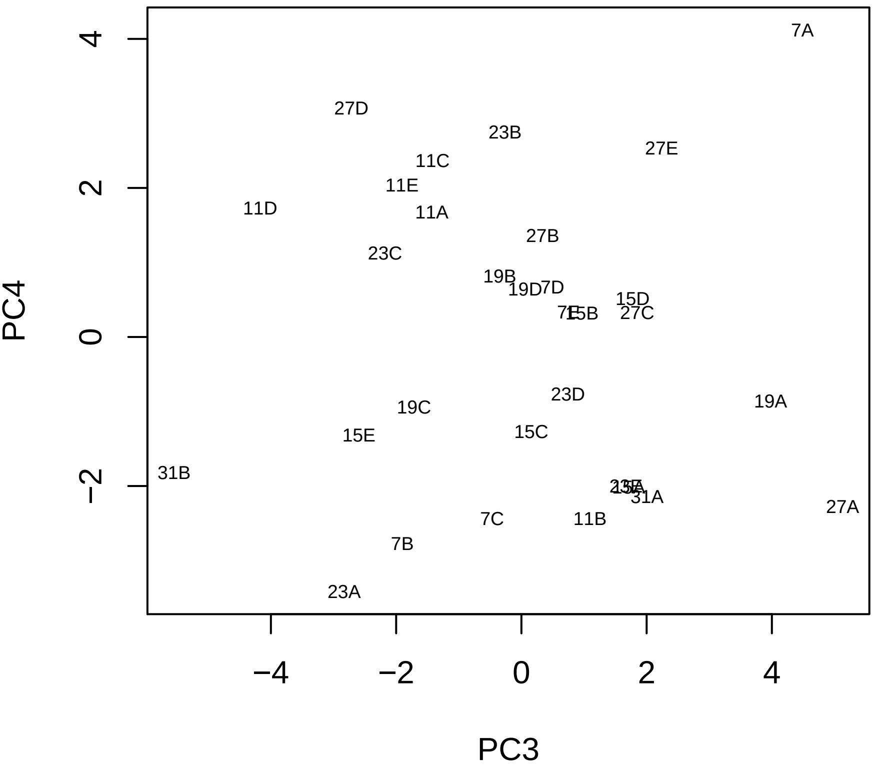

PC3
PC4
−4
−2
0
2
4
7A
27D
23B
27E
11C
11E
11D
11A
27B
23C
19B
19D
7D
15D
15B
27C
23D
19A
19C
15C
15E
31B
31A
27A
7C
11B
7B
23A

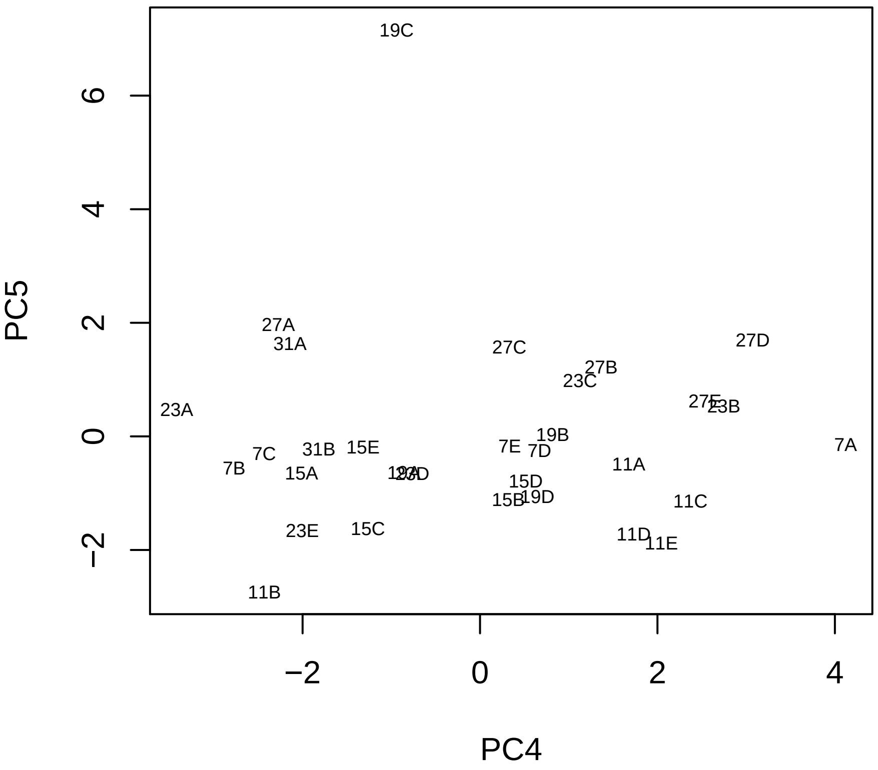

19C
27A
31A
27C
27D
27B
23C
23A
27E
23B
19B
7A
7E
7D
31B
15E
7C
7B
15A
11A
15D
15B
19D
11C
23E
15C
11D
11E
11B
6
4
2
0
−2
PC5
−2
0
2
4
PC4

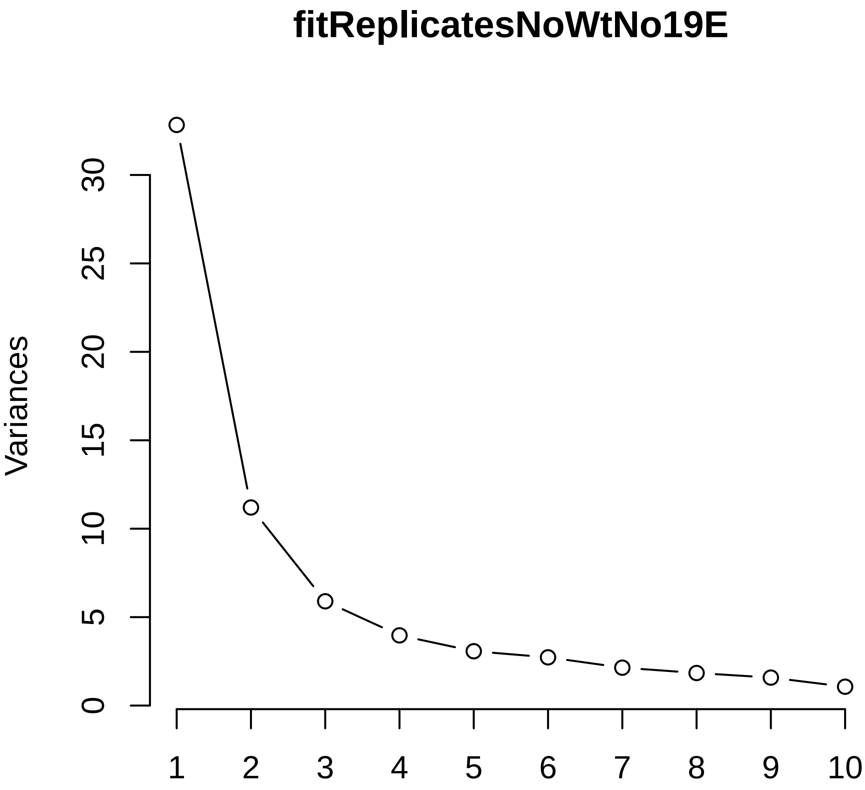

fitReplicatesNoWtNo19E
Variances
0
5
10
15
20
25
30
1
2
3
4
5
6
7
8
9
10
